# Functional Identification and Transcriptional Activity Analysis of *Dryopteris fragrans HMGR* Gene

**DOI:** 10.3390/plants14142190

**Published:** 2025-07-15

**Authors:** Meng Sun, Qian Ma, Xueqi Wang, Jialiang Guo, Jiaxuan Wang, Dongrui Zhang, Kirill Tkachenko, Wenzhong Wang, Ying Chang

**Affiliations:** 1 College of Life Sciences, Northeast Agricultural University, Harbin 150030, China; mengsun1999@163.com (M.S.); 18103654682@163.com (Q.M.); q15214670807@163.com (X.W.); guperman@163.com (J.G.); ngx1007372074@163.com (J.W.); dongruizhang96@gmail.com (D.Z.); 2 Peter the Great Botanical Garden, V.L. Komarov Botanical Institute of the Russian Academy of Sciences, Saint-Petersburg 197376, Russia; 3 Institute of Industrial Crops, Heilongjiang Academy of Agricultural Sciences, Harbin 150086, China

**Keywords:** *Dryopteris fragrans*, *HMGR*, gene function, promoter cloning

## Abstract

*Dryopteris fragrans* (L.) Schott synthesizes volatile sesquiterpenes through the mevalonate pathway (MVA), in which 3-hydroxy-3-methylglutaryl-CoA reductase (HMGR) serves as the key rate-limiting enzyme. Although HMGR plays a crucial role in terpenoid biosynthesis, its functional characteristics in *D. fragrans* and its involvement in stress responses remain unclear. This study identified three *HMGR* genes (*DfHMGR1/2/3*) from the transcriptome data of *D. fragrans*. Bioinformatics analysis revealed that the encoded proteins are localized to the endoplasmic reticulum and share high sequence similarity with fern homologs. Under abiotic stress conditions, *DfHMGR*s exhibited differential expression patterns, with marked upregulation under salt and drought stress. To validate the functions of these genes, we generated transgenic *Nicotiana tabacum* L. plants overexpressing *DfHMGR*s. Compared with wild-type controls, the transgenic lines showed enhanced tolerance to drought and heat stress. Promoter analysis identified functional regulatory regions controlling *DfHMGR* expression, and co-expression network analysis predicted 21 potential transcriptional regulators. This study validates the function of *D. fragrans HMGRs* in a heterologous system and provides candidate genes for improving stress resistance in plants.

## 1. Introduction

Terpenoids represent a major class of secondary metabolites, characterized by a general formula of (C5H8)n and structural diversity [1]. More than 80,000 terpenoids have been isolated, of which plant terpenoids are the most diverse and numerous, and the most widely studied [2]. Studies have confirmed that terpenoids maintain plant cell membrane integrity, resist insect pests and environmental stresses, participate in plant growth and development, and are components of the aromas and volatile oils produced by plants [3,4]. Terpenoids are widely used in medicine as analgesics and anti-malarials, in anti-tumor therapies, and in the treatment of diabetes and cardiovascular diseases, as well as in the preparation of natural food colorings, in the production of fragrances, and in biocontrol [5,6].

**Figure 1:**
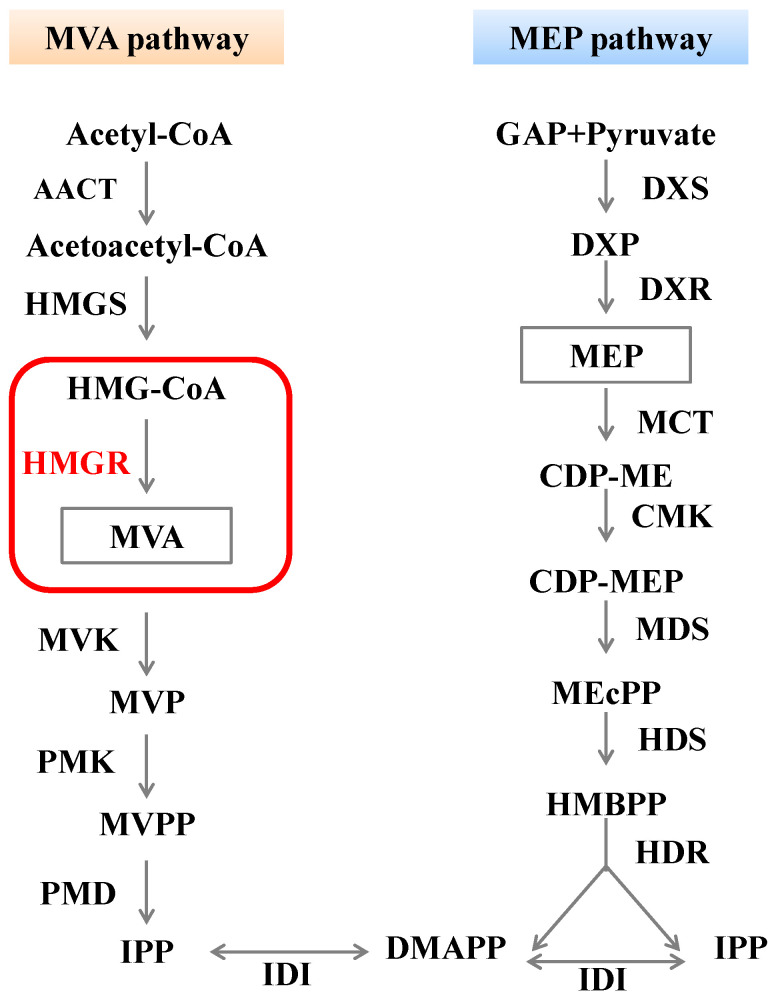
Schematic diagram of the MVA and MEP pathways in terpenoid biosynthesis. The left panel (orange background) shows the mevalonate (MVA) pathway; the right panel (blue background) displays the methylerythritol phosphate (MEP) pathway. The red box highlights HMGR, the key rate-limiting enzyme in the MVA pathway.

Plants produce terpenoids through two primary pathways (Figure 1): the MEP pathway, which occurs in the chloroplast, and the MVA pathway, which occurs in the cytoplasm [7]. In the MEP pathway, 1-Deoxy-D-Xylulose 5-Phosphate Synthase (DXS) catalyzes the conversion of pyruvate and D3P to DXP, and Deoxy-D-Xylulose 5-Phosphate Reductoisomerase (DXR) converts DXP to MEP. Due to the irreversibility of this reaction, DXS and DXR are considered to be the key enzymes in the MEP pathway [8]. Since 3-hydroxy-3-methylglutaryl-coenzyme A reductase (HMGR) is involved in catalyzing the first irreversible reaction of the MVA pathway and its activity is highly regulated, it is considered to be the essential rate-limiting enzyme in the MVA pathway [9].

Currently, *HMGR* genes have been cloned and identified from a variety of plants, including *Arabidopsis thaliana* (L.) Heynh. [10], *Salvia miltiorrhiza* Bunge [11], *Ginkgo biloba* L. [12], *Populus trichocarpa* Torr. & A.Gray ex Hook. [13], *Gossypium raimondii* Ulbr., *Gossypium arboreum* L., and *Gossypium hirsutum* L. [14], among numerous others. The 3-hydroxy-3-methylglutaryl-CoA reductase (HMGR) genes typically exist as multiple copies in plants, exhibiting remarkable phylogenetic and functional diversity [15]. Substantial variation in gene copy numbers has been observed across species [16]. These paralogous genes demonstrate distinct differentiation in both domain architecture and tissue-specific expression patterns [17,18]. Numerous studies have shown that HMGR expression levels have an impact on plant terpene content and phenotype; for example, researchers used Mevinolin as a competitive inhibitor, and inhibition of *HMGR* activity led to a reduction in the supply of IPP [19]. Hui Wei et al. [13] overexpressed *P. trichocarpa PtHMGR* in Nanlin895 poplar, resulting in elevated levels of several terpenoids. A study by Wang Shuai’s team elucidated the pivotal role of *Glycine max*. *GmHMGR* genes in plant growth and metabolic regulation [20]. The research demonstrated that *GmHMGR* genes participate in isoprenoid biosynthesis, a process intimately associated with plant growth and development. Heterologous overexpression of *GmHMGR* genes in *A. thaliana* accelerated plant development, with transgenic lines exhibiting significantly enhanced growth rates compared to wild-type plants. Furthermore, *GmHMGR* was shown to enhance salt stress tolerance, as transgenic plants displayed markedly improved stress tolerance. These findings systematically clarify the molecular mechanisms underlying *HMGR* genes’ functions in regulating plant growth and secondary metabolism.

*Dryopteris fragrans* is a plant of Dryopteridaceae, which grows in rock crevices after volcanic eruptions, and is most numerous in Wudalianchi and Mudanjiang in Heilongjiang Province. Wild-type *D. fragrans* morphological characteristics are presented in Figure S1. The secondary metabolites of *D. fragrans* are relatively abundant, and bioactive components such as m-phenyltriols, terpenoids, flavonoids, and phenylpropanoids have been isolated from *D. fragrans*, gradually revealing the diversity of *D. fragrans*’s chemical constituents. Studies have shown that *D. fragrans* is extremely effective in the treatment of a wide range of skin conditions such as dandruff, acne, and psoriasis [21,22]. In recent years, *HMGRs* have attracted much attention in the terpene synthesis pathway of seed plants and plant resistance to abiotic stresses. However, functional characterization of *HMGR* genes in fern species remains largely unexplored. This knowledge gap highlights the need for further investigation into the evolutionary conservation of *HMGR*-mediated stress adaptation mechanisms across plant lineages.

*D. fragrans* produces abundant sesquiterpenes through the mevalonate (MVA) pathway, yet the enzymatic regulation of this pathway remains poorly characterized. HMGR is the first rate-limiting enzyme in the MVA pathway, which is particularly important for the in-depth study of the terpene metabolism pathway of *D. fragrans*. In this study, we analyzed the expression patterns and functions of *DfHMGR*s and established a heterologous expression system—where ‘heterologous’ refers to functional gene transfer between evolutionarily distant species. We examined the stress tolerance of *N. tabacum* overexpressing *DfHMGR*s, and through cloning and functional analysis of *DfHMGR* gene promoters, investigated the gene-mediated stress response mechanisms in terpenoid metabolism.

## 2. Results

### 2.1. Bioinformatics Analysis of DfHMGRs

We first obtained the *A. thaliana* AtHMGR protein sequences through the NCBI (HMGR1:NP_177775.2, HMGR2:NP_179329.1), and used Tbtools -II (Toolbox for Biologists) v2.026 to obtain three DfHMGR proteins with high similarity to the AtHMGRs in the *D. fragrans* transcriptome database, with the gene numbers comp35287, comp25251, and comp38904 named *DfHMGR1*, *DfHMGR2,* and *DfHMGR3,* respectively.

After prediction, all three genes were found to have complete open reading frames. The basic physicochemical properties were analyzed by online prediction with the ExPASy ProtParam tool (https://web.expasy.org/protparam/, accessed on 27 September 2023) [23] (Table 1). The predicted secondary structures of DfHMGR1–3 proteins are presented in Supplementary Figure S2. Secondary structure analysis revealed that DfHMGR1–3 proteins exhibit characteristic alpha–beta topology, with α-helices (45.44–46.19%, 259–267 aa) constituting the predominant secondary structure element, followed by extended strands (14.49–16.26%, 83–94 aa), random coils (31.49–34.74%, 182–198 aa), and β-turns (5.09–6.06%, 29–35 aa). The high α-helical content and remarkable structural conservation implies these proteins likely contain transmembrane domains and share conserved catalytic functions in terpenoid biosynthesis pathways. Homology modeling using the SEISS-MODEL system generated high-quality structures for DfHMGR1 (template A9SYW2.1.A, 70.92% sequence identity, GMQE = 0.84), DfHMGR2 (A0A0D6R8C6.1.A, 72.56%, GMQE = 0.83), and DfHMGR3 (A0A2R6XGR7.1.A, 71.68%, GMQE = 0.83). The tertiary structures of DfHMGR1–3 are presented in Figure S3. Their conserved spatial configurations further support functional similarities among DfHMGR1–3 proteins. Predictive analysis of the subcellular localization of DfHMGR proteins using an online prediction website (http://www.softberry.com/, accessed on 25 October 2023) revealed that all DfHMGR proteins are localized to the endoplasmic reticulum [24,25,26].

**Figure 2:**
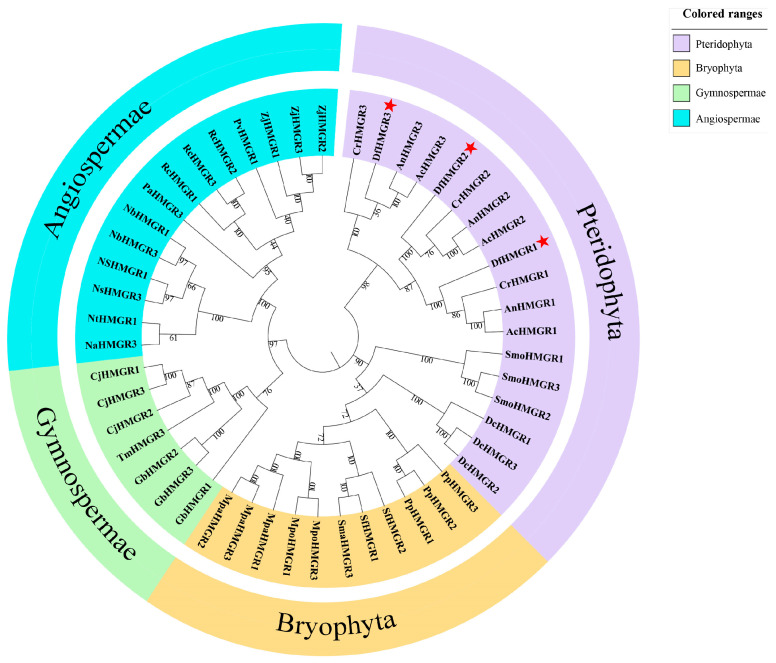
Phylogenetic relationships of HMGR proteins. The circular phylogenetic tree displays four plant lineages by colored sectors, light purple for Pteridophyta, pale yellow for Bryophyta, light green for Gymnospermae, and light blue for Angiospermae, with red stars indicating DfHMGR1–3 proteins (full names provided in Supplementary Table S1).

To explore the affinities of DfHMGRs with HMGR from other species, we performed amino acid sequence comparisons and constructed a phylogenetic tree (Figure 2). Taxonomic classification and protein nomenclature of HMGR sequences are detailed in Table S1. DfHMGRs are located in the same branch as the HMGRs of *Adiantum nelumboides* X. C. Zhang, *Ceratopteris richardii* Brongn., and *Adiantum capillus-veneris* L. DfHMGRs showed >85% sequence similarity with HMGRs from *A. nelumboides*, *C. richardii*, and *A. capillus-veneris*, whereas the three DfHMGR isoforms (DfHMGR1–3) shared 77.82% similarity with high conservation. Sequence alignment results are presented in Figure S4.

### 2.2. DfHMGR Cloning and DfHMGR Induction

Total RNA was extracted from *D. fragrans* and reverse-transcribed to obtain cDNA sequences, followed by cloning of the coding sequences (CDSs) for three target genes. The complete CDSs of *DfHMGR1–3* are provided in Supplementary Table S2. The full lengths of *DfHMGR1*, *DfHMGR2*, and *DfHMGR3* genes were determined to be 1722 bp, 1737 bp, and 1713 bp, respectively, consistent with our internal transcriptomic analyses.

The recombinant plasmids carrying *DfHMGR1–3* genes were transformed into *Escherichia coli* BL21-Star™ (DE3)-competent cells (Shanghai Weidi Biotechnology Co., Ltd., Shanghai, China). Protein expression was induced at OD_600_ = 0.6 under optimized conditions: DfHMGR1: 0.5 mM isopropyl β-D-1-thiogalactopyranoside (IPTG), 37 °C, 150 rpm, 4 h; DfHMGR2: 1.0 mM IPTG, 37 °C, 150 rpm, 2 h; and DfHMGR3: 0.5 mM IPTG, 37 °C, 150 rpm, 4 h (complete sequences in Supplementary Table S3). Soluble expression was confirmed by SDS-PAGE (Supplementary Figure S5).

### 2.3. Subcellular Localization of DfHMGRs

**Figure 3:**
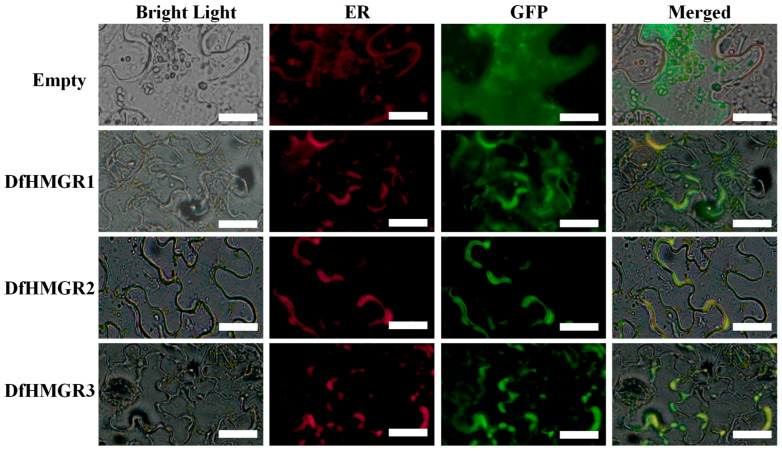
Subcellular localization of DfHMGRs in *N. benthamiana* leaf epidermal cells. The figure shows four experimental groups (empty vector, DfHMGR1, DfHMGR2, and DfHMGR3) imaged under four channels: Bright Light (cell morphology), ER (red fluorescence, ER marker), GFP (green fluorescence), and merged channels (yellow indicates co-localization). Scale bars = 20 μm.

The recombinant plasmids pCAMBIA2300-*DfHMGR1*-EGFP, pCAMBIA2300-*DfHMGR2*-EGFP, and pCAMBIA2300-*DfHMGR3*-EGFP were co-transformed with an endoplasmic reticulum (ER) marker plasmid into *Agrobacterium rubi* LBA4404-competent cells (purchased from Shanghai Weidi Biotechnology Co., Ltd., Shanghai, China) and transiently expressed in *Nicotiana benthamiana* Domin leaves. Confocal microscopy confirmed that DfHMGR1, DfHMGR2, and DfHMGR3 were localized to the ER, consistent with bioinformatic predictions (Figure 3). The figure displays four treatments (empty vector, DfHMGR1, DfHMGR2, and DfHMGR3) imaged under bright field, ER (red), GFP (green), and merged channels (yellow co-localization), with uniform scale bars.

### 2.4. Analysis of DfHMGR Expression Patterns

**Figure 4:**
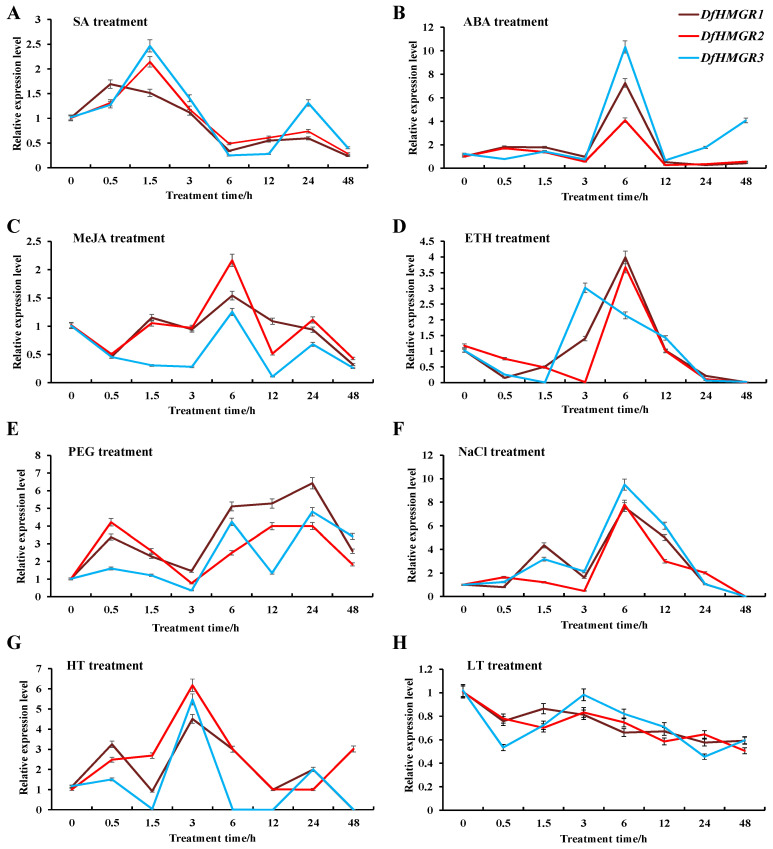
Relative expression levels of *DfHMGR*s. (**A**–**D**) Different hormone treatments: SA (salicylic acid), ABA (abscisic acid), MeJA (methyl jasmonate), and ETH (ethephon). (**E**–**H**) Different abiotic stress treatments: PEG (drought stress), NaCl (salt stress), HT (high temperature), and LT (low temperature). Data represent mean ± SEM (*n* = 3) of three biological replicates, normalized to *Df18S* reference genes. Relative expression levels were calculated using the 2^−ΔΔCt^ method. Brown, red, and blue lines denote *DfHMGR1*, *DfHMGR2,* and *DfHMGR3*. X-axis: treatment time (h); Y-axis: relative expression level (fold change vs. 0 h control).

We systematically investigated the expression profiles of *DfHMGR* genes under various hormonal treatments (Figure 4A–D) including salicylic acid (SA), abscisic acid (ABA), methyl jasmonate (MeJA), and ethephon (ETH). Quantitative analysis revealed distinct temporal expression patterns among the three *DfHMGR* isoforms. SA treatment elicited a biphasic response characterized by initial upregulation followed by gradual decline across all isoforms (Figure 4A). The most dramatic induction was observed under ABA treatment (Figure 4B), with expression levels peaking at 6 h post-treatment (7.15-fold for *DfHMGR1*, 3.98-fold for *DfHMGR2,* and 8.45-fold for *DfHMGR3* compared to 0 h controls). MeJA treatment resulted in sustained upregulation (Figure 4C), reaching maximum levels at 6 h (1.51-fold for *DfHMGR1*, 2.13-fold for *DfHMGR2,* and 1.25-fold for *DfHMGR3*). ETH responses exhibited isoform-specific kinetics (Figure 4D), with *DfHMGR1* and *DfHMGR2* showing peak expression at 6 h while *DfHMGR3* reached maximal levels earlier at 3 h.

The abiotic stress response analysis (Figure 4E–H) revealed that all three *DfHMGR* isoforms exhibited differential expression patterns under various stress conditions. Under PEG-6000-induced drought stress (Figure 4E), the isoforms showed characteristic temporal expression fluctuations with peak induction at intermediate time points. NaCl treatment (Figure 4F) induced rapid and sustained upregulation of all isoforms, with *DfHMGR2* demonstrating the strongest response (9.48-fold at 6h). High-temperature stress (Figure 4G) resulted in consistent but moderate upregulation (4.5–6.17-fold across isoforms), whereas low-temperature treatment (Figure 4H) elicited minimal expression changes (<1.2-fold variation), indicating temperature-dependent regulatory mechanisms.

### 2.5. Stress Tolerance Enhanced by Overexpression of DfHMGRs in N. tabacum

#### 2.5.1. Overexpressing *DfHMGR*s Improve Salinity Tolerance in *N. tabacum* Seeds

To investigate the functional roles of *DfHMGR*s, we generated recombinant plasmids (2300-35S-AD-*DfHMGR1*-nos, 2300-35S-AD-*DfHMGR2*-nos, and 2300-35S-AD-*DfHMGR3*-nos) and transformed them into *A. rubi* LBA4404-competent cells. After activation, the bacterial suspension was used to infect 3-day pre-cultured *N. tabacum* leaf disks, followed by 3 d of co-cultivation. The explants were then transferred to kanamycin (Kan)-containing selection medium until dark green resistant shoots emerged. Subsequently, the resistant shoots were transferred to rooting medium supplemented with 50 mg/L Kan, and well-rooted plants were transplanted into nutrient soil. The resulting transgenic *N. tabacum* plants are depicted in Supplementary Figure S6. In total, twenty-four independent transgenic lines overexpressing *DfHMGR1*, *DfHMGR2*, or *DfHMGR3* were obtained. Genomic DNA was extracted from mature *N. tabacum* leaves and subjected to PCR amplification using vector-specific primers (2300-35S-AD-nos), which yielded an 857 bp target band (Supplementary Figure S7), consistent with the expected size. No amplification product was detected in wild-type *N. tabacum*, whereas single specific bands matching the positive control were observed in *DfHMGR1* transgenic lines (#1–#6), *DfHMGR2* transgenic lines (#1–#7), and *DfHMGR3* transgenic lines (#1–#3, #5, #6, #8).

We identified homozygous T2 lines by PCR and 3:1 segregation ratio analysis, then obtained T3 seeds through self-pollination, with homozygosity further confirmed by a 100% survival rate at the four-leaf stage under selection. Both T3 transgenic and wild-type seeds were germinated on 1/8 MS medium containing 0, 50, or 100 mM NaCl, with germination rates and phenotypic variations recorded at the four-leaf stage.

**Figure 5:**
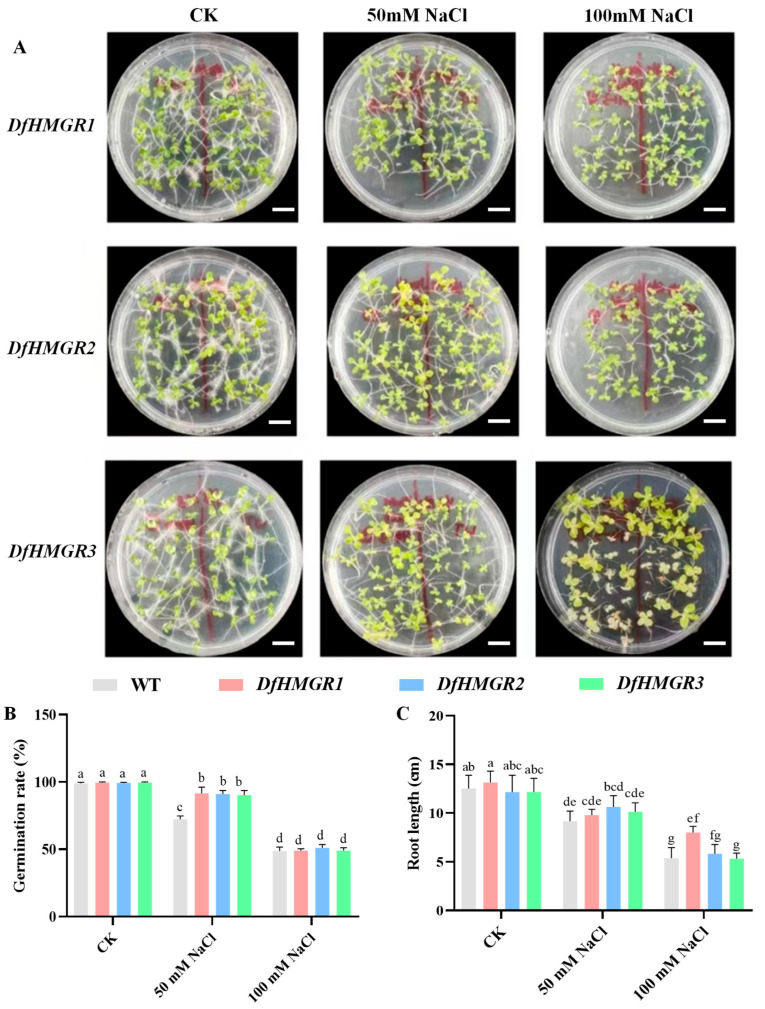
Salt stress responses of *DfHMGR*-overexpressing *N. tabacum* seeds. (**A**) Germination phenotypes under CK (0 mM NaCl), 50 mM NaCl, and 100 mM NaCl treatments. The red demarcation line separates transgenic seeds (left, *DfHMGR1–3*) from wild-type seeds (WT, right). Scale bars = 1 cm (for all panels). (**B**) Germination rates (gray: WT; red: *DfHMGR1*; blue: *DfHMGR2*; green: *DfHMGR3*). (**C**) Root length measurements (color codes correspond to (**B**)). Data represent mean ± SD (*n* = 3 biological replicates, 30 seeds each). Different lowercase letters indicate statistically significant differences (*p* < 0.05, one-way ANOVA with Tukey’s test). CK: untreated control (0 mM NaCl); WT: wild type.

As shown in Figure 5, distinct growth differences were observed between the wild type (WT) and three transgenic lines (*DfHMGR1/2/3*) under salt stress. The plate photographs (Figure 5A) clearly showed that at 50 mM NaCl treatment, *DfHMGR1*, *DfHMGR2,* and *DfHMGR3* lines exhibited better growth performance than the WT, with more robust root systems. Quantitative analysis (three replicates per group, 30 seeds each) revealed that under 50 mM NaCl condition, the germination rates of *DfHMGR1*, *DfHMGR2,* and *DfHMGR3* were 1.26-fold (*p* < 0.05), 1.26-fold (*p* < 0.05), and 1.25-fold (*p* < 0.05) higher than WT, respectively, while no significant difference in root length was detected between transgenic lines and the WT. Notably, when the NaCl concentration increased to 100 mM, the germination rates of all experimental groups decreased compared to the 50 mM treatment, with no significant intergroup differences observed. At this higher concentration, only *DfHMGR1* showed a moderate advantage in root length over WT (*p* < 0.05), whereas *DfHMGR2* and *DfHMGR3* displayed no significant difference compared to WT.

#### 2.5.2. Overexpressing *DfHMGR* Improves *N. tabacum* Resistance to Drought and Heat

**Figure 6:**
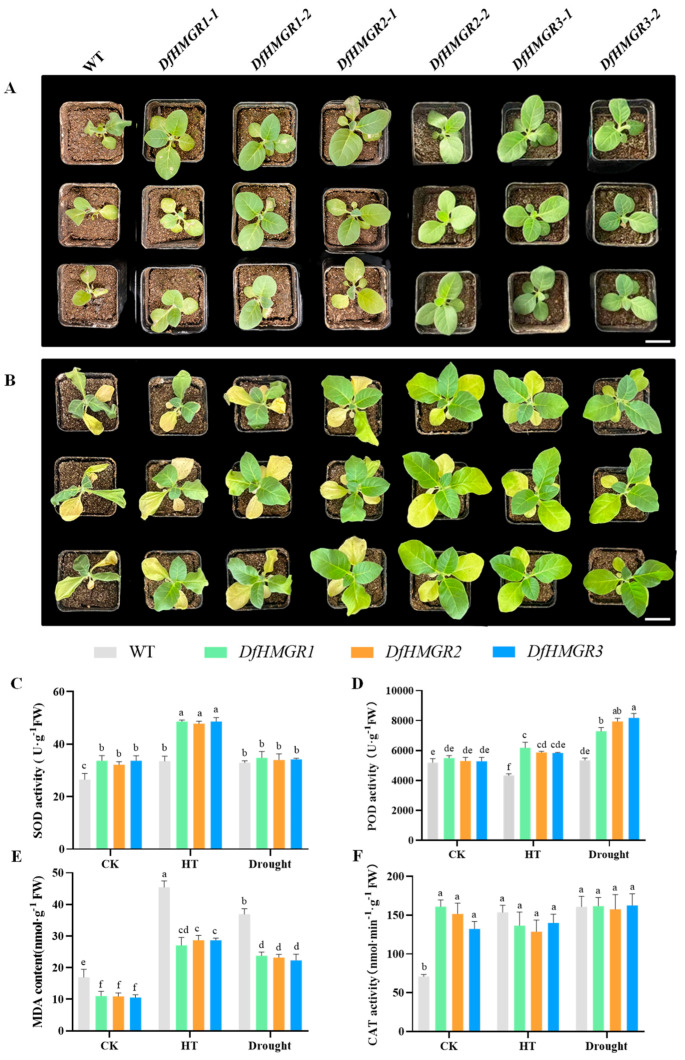
Thermotolerance and drought resistance analysis of *DfHMGR*-overexpressing *N. tabacum* plants. (**A**) Phenotypes under 42 °C heat stress (three rows represent three biological replicates): wild type (WT), *DfHMGR1-1* and *DfHMGR1-2* (two independent transgenic lines of *DfHMGR1*), *DfHMGR2-1* and *DfHMGR2-2* (two independent lines of *DfHMGR2*), and *DfHMGR3-1* and *DfHMGR3-2* (two independent lines of *DfHMGR3*). Scale bars = 1 cm. (**B**) Phenotypes under drought stress (arrangement and replicates same as (**A**)). Scale bars = 1 cm. (**C**–**F**) Quantitative analysis of physiological parameters (gray: WT; red: *DfHMGR1*; blue: *DfHMGR2*; green: *DfHMGR3*), with data presented as mean ± SD (*n* = 3 biological replicates): (**C**) SOD activity, (**D**) POD activity, (**E**) MDA content, and (**F**) CAT activity. Different lowercase letters indicate statistically significant differences (*p* < 0.05, one-way ANOVA with Tukey’s test). SOD: superoxide dismutase; POD: peroxidase; MDA: malondialdehyde; CAT: catalase; CK: untreated control (normal condition); HT: high temperature; Drought: drought treatment.

Following the generation of *DfHMGR*-overexpressing transgenic *N. tabacum* lines, we conducted parallel stress assays involving 42 °C heat shock and progressive drought to characterize gene function. Quantitative analysis revealed that after 4 h heat exposure, transgenic lines maintained high viability compared to wild-type (WT) plants, which exhibited pronounced wilting. Under 14-day drought stress, WT plants showed ~60% leaf wilting area, whereas transgenic lines retained >70% leaf integrity (Figure 6A,B).

Physiological measurements of wild-type (WT) and *DfHMGR*-overexpressing transgenic *N. tabacum* under three conditions (room temperature, 42 °C for 4h, natural drought) showed the following: under normal conditions, transgenic lines had higher SOD activity than the WT (*p* < 0.05); under heat stress, transgenic lines showed greater SOD activity increases (*p* < 0.05), while no significant difference was observed under drought stress (*p* > 0.05). POD activity showed no genotypic difference under control conditions (*p* > 0.05), but was higher in transgenic lines under stress (*p* < 0.05). MDA content was consistently lower in transgenic lines across all treatments (*p* < 0.05). CAT activity was higher in transgenic lines at baseline (*p* < 0.05), but showed no significant changes under stress (*p* > 0.05) (Figure 6C–F).

### 2.6. Identification of DfHMGR Promoter Active Sites

**Figure 7:**
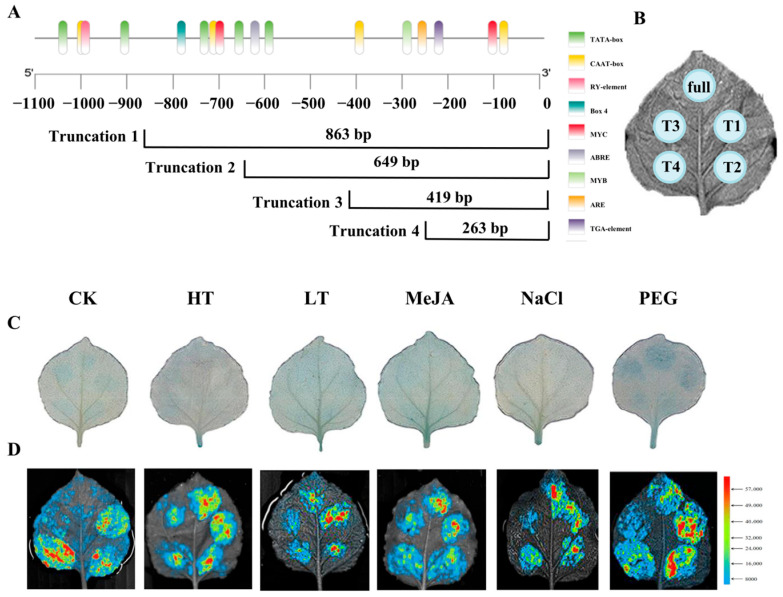
Functional characterization and active site identification of *DfHMGR1* promoter. (**A**) Schematic diagram of *DfHMGR1* promoter region showing four truncated fragments (T1: 863 bp; T2: 649 bp; T3: 419 bp; T4: 263 bp) from 5′- to 3′-end. Cis-acting elements are color-coded as dark green = TATA-box (core promoter), yellow = CAAT-box (enhancer), pink = RY-element (GA-inducible), cyan = Box 4 (light-responsive), red = MYC (jasmonate signaling), lavender gray = ABRE (ABA-responsive), light green = MYB (transcription factor binding), orange = ARE (anaerobic-responsive), and purple = TGA-element (hormone/drought response). (**B**) Schematic representation of transient expression system in *N. benthamiana* leaves, indicating expression regions of full-length promoter (full) and four truncated fragments (T1–T4). (**C**) GUS stain to identify the *DfHMGR1* promoter active site under different treatments. (**D**) Dual-luciferase reporter assay to identify *DfHMGR1* promoter active site with color scale indicating relative luciferase activity. CK: untreated control; HT: high temperature; LT: low temperature; MeJA: methyl jasmonate; NaCl: salt stress; PEG: drought stress.

**Figure 8:**
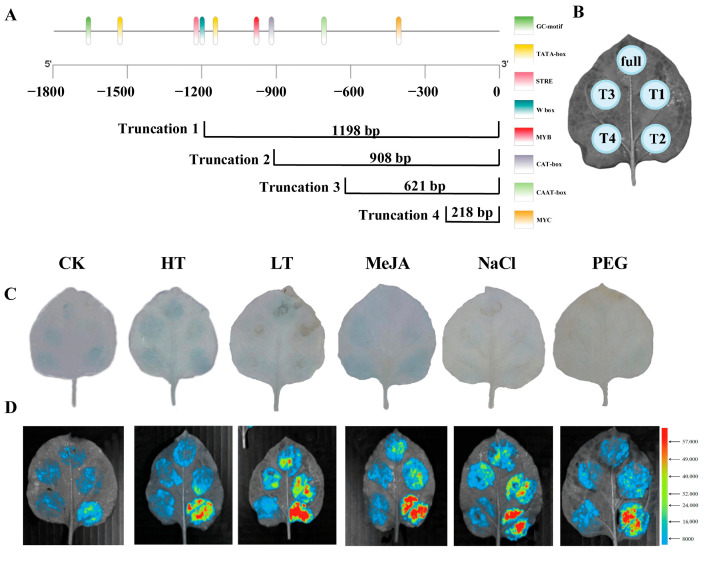
Functional characterization and active site identification of *DfHMGR2* promoter. (**A**) Schematic diagram of *DfHMGR2* promoter region showing four truncated fragments (T1: 1198 bp; T2: 908 bp; T3: 621 bp; T4: 218 bp) from 5′- to 3′-end. Cis-acting elements are color-coded as dark green = GC-motif (hypoxia-responsive), yellow = TATA-box (core promoter), pink = STRE (stress-responsive), cyan = Wbox (drought-responsive), red = MYB (transcription factor binding), lavender gray = CAT-box (meristem-specific), light green = CAAT-box (enhancer), and orange = MYC (jasmonate signaling). (**B**) Schematic representation of transient expression system in *N. benthamiana* leaves, indicating expression regions of full-length promoter (full) and four truncated fragments (T1–T4). (**C**) GUS stain to identify the *DfHMGR2* promoter active site under different treatments. (**D**) Dual-luciferase reporter assay to identify *DfHMGR2* promoter active site with color scale indicating relative luciferase activity. CK: untreated control; HT: high temperature; LT: low temperature; MeJA: methyl jasmonate; NaCl: salt stress; PEG: drought stress.

**Figure 9:**
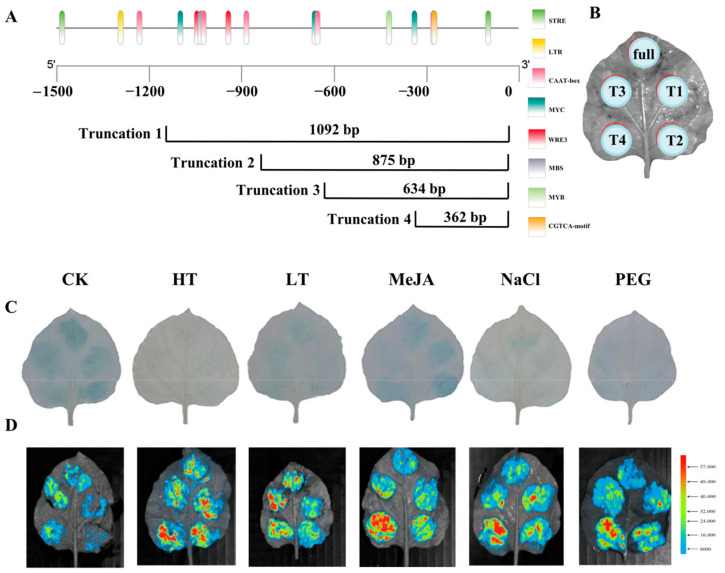
Functional characterization and active site identification of *DfHMGR3* promoter. (**A**) Schematic diagram of *DfHMGR3* promoter region showing four truncated fragments (T1: 1092 bp; T2: 875 bp; T3: 634 bp; T4: 362 bp) from 5′- to 3′-end. Cis-acting elements are color-coded as dark green = STRE (stress-responsive), yellow = LTR (cold-responsive), pink = CAAT-box (enhancer), cyan = MYC (jasmonate signaling), red = WRE3 (transcription factor binding), lavender gray = MBS (drought-responsive), light green = MYB (transcription factor binding), and orange = CGTCA-motif (MeJA-responsive). (**B**) Schematic representation of transient expression system in *N. benthamiana* leaves, indicating expression regions of full-length promoter (full) and four truncated fragments (T1–T4). (**C**) GUS stain to identify the *DfHMGR3* promoter active site under different treatments. (**D**) Dual-luciferase reporter assay to identify *DfHMGR3* promoter active site with color scale indicating relative luciferase activity. CK: untreated control; HT: high temperature; LT: low temperature; MeJA: methyl jasmonate; NaCl: salt stress; PEG: drought stress.

To investigate the activity of *DfHMGR* promoters, we performed chromosome walking from the CDSs upstream at the genomic level using FPNI-PCR with specific primers (FP1-16). This approach successfully cloned the promoter sequences of *DfHMGR1* (1088 bp), *DfHMGR2* (1548 bp), and *DfHMGR3* (1496 bp). Bioinformatics analysis using Plant Care identified multiple cis-acting elements in these promoters, including core elements (CAAT-box and TATA-box) and various functional elements (Figure 7A, Figure 8A and Figure 9A). The *DfHMGR1* promoter contains the following functional elements: RY-element (GA-inducible and anther-specific), Box 4 (light-responsive), MYC (jasmonate signaling via MYB binding), ABRE (ABA-responsive), MYB (transcription factor binding), ARE (anaerobic-responsive), and TGA-element (responsive to hormones, drought, and cold). The *DfHMGR2* promoter includes the GC-motif (hypoxia-responsive), STRE (stress-responsive), W box (drought-responsive), MYB (transcription factor binding), CAT-box (meristem-specific), and MYC (jasmonate signaling via MYB binding). The *DfHMGR3* promoter comprises STRE (stress-responsive), LTR (cold-responsive), MYC (jasmonate signaling via MYB binding), WRE3 (transcription factor binding), MBS (drought-responsive), MYB (transcription factor binding), and CGTCA-motif (MeJA-responsive).

To characterize promoter activity, we generated five fragments for each promoter (full-length plus four truncations: Truncation 1–Truncation 4), deliberately excluding regions containing phytohormone-, abiotic stress-, and light-responsive elements. The truncation strategy avoided potential interference from these response elements while maintaining core promoter functionality.

Figure 7B, Figure 8B and Figure 9B displays the transient transformation patterns in *N. benthamiana*. leaves after *Agrobacterium*-mediated introduction of recombinant plasmids containing *DfHMGR* promoter fragments fused to pBI121-EGFP-GUS and pGreen II-0800-luc vectors.

The integrated analysis in Figure 7C,D, Figure 8C,D and Figure 9C,D combining quantitative LUC reporter assays with qualitative GUS staining, highlights functional diversification across *DfHMGR* promoter family members. The *DfHMGR1* promoter exhibits exclusive drought stress responsiveness, supported by robust LUC signals and GUS staining under PEG treatment. The *DfHMGR2* promoter harbors modular cis-elements that coordinate responses to drought, cold, MeJA, and salt stresses, with activation patterns consistently captured by both LUC and GUS assays. For *DfHMGR3*, a fragment-specific regulatory mechanism emerges, where distinct truncated regions (T1–T4) mediate stress-specific responses, as confirmed by congruent LUC quantification and GUS spatial expression.

### 2.7. Prediction of DfHMGR Gene Expression Regulating Transcription Factors

**Figure 10:**
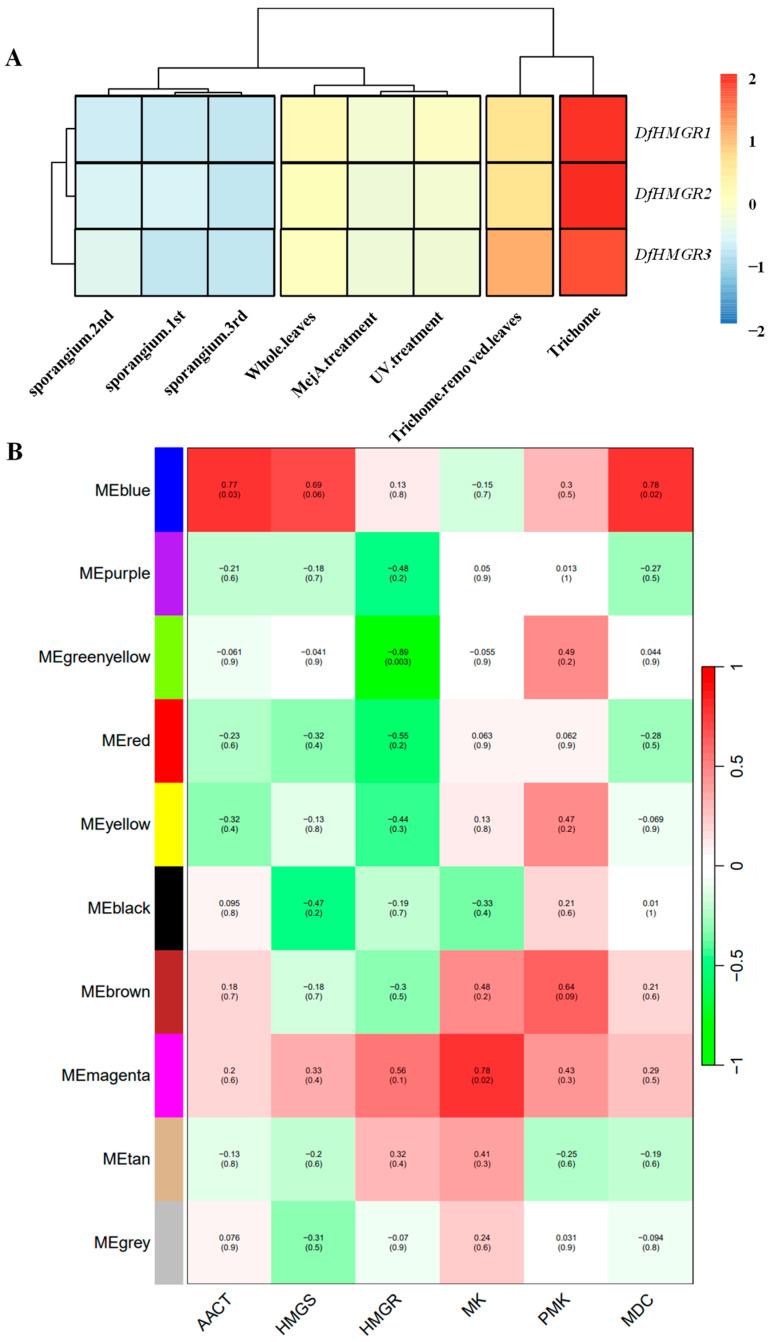
Co-expression network analysis of *DfHMGR* regulatory transcription factors. (**A**) Hierarchical clustering of *DfHMGR1–3* expression profiles across developmental and treatment conditions. Heatmap colors (blue to red) represent normalized expression levels (−2 to 2) in sporangia at three developmental phases (1st–3rd), whole leaves, MeJA-treated leaves, UV-treated leaves, trichome-removed leaves, and trichomes. (**B**) Weighted gene co-expression network analysis showing Pearson correlations (coefficients with *p*-values in parentheses) between MVA pathway genes (*AACT*, *HMGS*, *HMGR*, *MK*, *PMK*, and *MDC*) and identified gene modules (MEblue to MEgrey).

Component prediction showed that the promoter of the *DfHMGR* gene contained binding sites for MYB transcription factors in response to the jasmonate signaling pathway (Figure 7A, Figure 8A and Figure 9A). Figure 10A clearly visually represents the expression patterns of *DfHMGR1*, *DfHMGR2*, and *DfHMGR3* across eight different sample types. Notably, the three genes exhibit highly consistent expression trends: they are all strongly upregulated (red) in trichomes, while showing low expression (blue) in sporangium at different developmental stages (1st/2nd/3rd) and trichome-removed leaves. Furthermore, their expression levels display synchronous variations in whole leaves and MeJA-treated and UV-treated samples. This coordinated expression pattern indicates that *DfHMGR1*, *DfHMGR2*, and *DfHMGR3* may be regulated by similar transcriptional mechanisms and potentially participate in related biological functions in *D. fragrans*. To elucidate this regulation, we performed co-expression network analysis identifying the Magenta module’s strong association with *DfHMGR*s (Figure 10B). Screening 570 Magenta module genes via GFAP (Gene Functional Annotation for Plants)v1.0.0 software yielded 21 putative regulatory transcription factors. Detailed molecular characteristics of these candidates are provided in Supplementary Table S4.

## 3. Discussion

HMGR is a key rate-limiting enzyme in the terpenoid synthesis pathway–MVA pathway, which is of great importance for the regulation of terpenoid synthesis in plants. Plant HMGRs are encoded by the *HMGR* gene family, and different HMGRs regulate the synthesis of different functional metabolites in the MVA pathway [27]. For example, *HMGR1* in *Solanum tuberosum* L. has a role in steroid synthesis in response to mechanical damage [28]. In *Persea americana* Mill. cv. Hass, reduced HMGR activity in small-fruit variants is directly associated with compromised fruit growth [29]. Current research on *HMGR* has focused on terpene synthesis pathways in seed plants and plant stress tolerance studies, but the function of *HMGR* has not yet been verified in ferns. The terpene metabolism of *D. fragrans* is relatively vigorous, and it can emit a large number of aromatic compounds, mainly sesquiterpenes, which are produced by the MVA pathway, and the HMGR protein plays an important role as a key rate-limiting enzyme, which is probably related to the resilience of *D. fragrans* [30]. Therefore, we investigated the *DfHMGR* gene family of *D. fragrans*, which encodes HMGR proteins, to further our understanding of terpene metabolism in *D. fragrans*. We first cloned the *DfHMGR*s of *D. fragrans*, constructed an *N. tabacum* plant overexpressing *DfHMGRs,* and tested the resistance of the transgenic strain, which provides an important basis for further interpretation of the function of the *DfHMGR*s of *D. fragrans*.

Accumulating evidence indicates that the HMGR gene family encodes evolutionarily conserved rate-limiting enzymes in the plant mevalonate pathway. Studies on *A. thaliana* demonstrate that LerHMGR1 and LerHMGR2 isoforms exhibit distinct catalytic efficiencies for HMG-CoA-to-mevalonate conversion [31]. Phenotypic analyses of *HMGR1* mutants reveal coordinated developmental defects including growth retardation, premature senescence, and complete male sterility, associated with disrupted sterol biosynthesis [32]. While endoplasmic reticulum localization predominates, emerging evidence suggests compartmentalized distribution—notably PgHMGR’s dual ER/peroxisome localization [33]. Our investigation of *D. fragrans* HMGRs (DfHMGR1–3) reveals conserved functional features. Phylogenetic analysis (Figure 2) clusters DfHMGRs with fern homologs from *A. nelumboides*, *C. richardii*, and *A. capillus-veneris* (>85% sequence similarity), indicating potential functional conservation in pteridophytes. Confocal microscopy (Figure 3) visually confirms ER localization for all three DfHMGR isoforms, showing clear co-localization with ER markers. These findings provide experimental evidence for understanding terpenoid metabolism regulation in fern species.

Building on these conserved features, we systematically analyzed the expression patterns of *DfHMGR*s under hormonal and abiotic stress treatments (Figure 4). *DfHMGR*s exhibited distinct expression patterns under ABA and MeJA treatments, with *DfHMGR3* showing the strongest upregulation under ABA induction (8.45-fold), which may suggest their participation in ABA-mediated stress responses, while *DfHMGR2* responded more strongly to MeJA (2.13-fold), implying that different isoforms may participate in distinct hormone signaling pathways. Under abiotic stresses, salt stress (NaCl) strongly induced the expression of all *DfHMGR*s, particularly *DfHMGR2* (9.48-fold), which may indicate its potential key role in osmotic regulation and demonstrate isoform-specific functions in stress resistance. High-temperature (HT) treatment resulted in upregulation across all isoforms (4.5–6.17-fold), with *DfHMGR3* showing the highest induction, possibly related to thermotolerance functions. In contrast, low temperature (LT) caused only minimal changes (<1.2-fold), indicating temperature-dependent regulation of *DfHMGR*s. The biphasic response (initial increase followed by decline) induced by SA and ETH treatments, along with isoform-specific temporal dynamics (e.g., *DfHMGR3* peaking at 3h under ETH), further support the functional divergence of *DfHMGR*s in stress adaptation, although the precise regulatory mechanisms require further investigation.

This study employed *N. tabacum* as a heterologous expression system based on the evolutionarily conserved catalytic function of HMGR as the rate-limiting enzyme in the mevalonate (MVA) pathway across land plants. The enzyme maintains highly conserved substrate (HMG-CoA) specificity and product (MVA) generation characteristics from ferns to angiosperms. Previous studies have confirmed the functional conservation of *HMGR* genes in heterologous systems. For instance, *A. thaliana* AtHMGR is post-translationally regulated by protein phosphatase 2A (PP2A) to mediate responses to various environmental stresses [34]. Furthermore, heterologous overexpression of soybean *GmHMGR* in *A. thaliana* significantly enhances host resistance to multiple stresses [20]. Although *HMGR* is broadly involved in plant stress response regulation, our study reveals that *D. fragrans HMGR* genes display a specific response pattern to salt and drought stresses. This observation not only confirms the conserved core functions of HMGR proteins but also suggests unique regulatory mechanisms that may have evolved in ferns to adapt to extreme habitats. These evolutionary and functional precedents justified our approach to characterize *DfHMGR*s through heterologous expression in *N. tabacum*, as detailed in the following stress response analyses. Functional validation experiments demonstrated that *DfHMGR* overexpression enhanced salt stress tolerance in transgenic *N. tabacum* (Figure 5). Under 50 mM NaCl conditions, all transgenic lines showed higher germination rates compared to the wild type (26%, 26%, and 25% increase for *DfHMGR1*, *DfHMGR2,* and *DfHMGR3,* respectively; *p* < 0.05), which correlated well with the high expression level of *DfHMGR2* under salt stress. However, at 100 mM NaCl, only *DfHMGR1* lines displayed root length advantage (15% longer than WT; *p* < 0.05), possibly indicating functional divergence among isoforms under extreme stress conditions.

The results from Figure 6 further confirmed the protective roles of *DfHMGR*s under heat and drought stresses. Transgenic plants exhibited approximately 50% higher SOD activity at 42 °C (*p* < 0.05), consistent with the SOD-mediated antioxidant defense mechanism reported in transgenic *N. tabacum* [35]. Concurrently, *DfHMGR*s induced a significant increase in POD activity (*p* < 0.05) under stress conditions, effectively reducing hydrogen peroxide accumulation through a mechanism analogous to that observed in transgenic groundnut [36]. The markedly reduced MDA content (30% lower than WT; *p* < 0.05) indicated effective suppression of lipid peroxidation by *DfHMGR*s, corroborating findings in cold-stressed *N. tabacum* regarding MDA content changes [37]. It should be noted that CAT activity showed no significant alteration (*p* > 0.05), suggesting *DfHMGR*s primarily regulate ROS scavenging through the SOD-POD pathway rather than the CAT system. These findings demonstrate that *DfHMGR* expression can maintain cell membrane stability and reduce cellular damage caused by free radicals, thereby improving plant resistance to high temperatures and drought. Future studies should focus on establishing a genetic transformation system for *D. fragrans* and generating *DfHMGR*-overexpressing transgenic plants to further validate the functions of *DfHMGR*s in *D. fragrans*.

The promoter regions of *HMGR* genes contain various cis-acting elements involved in biotic and abiotic stress responses. For instance, multiple ETH-, MeJA-, and SA-responsive cis-elements have been identified in the promoters of apple HMGR gene family members, with *MdHMGR2* and *MdHMGR4* expression being markedly induced by these hormones [38,39]. In this study, we successfully cloned the promoter sequences of *DfHMGR1* (1088 bp), *DfHMGR2* (1548 bp), and *DfHMGR3* (1496 bp) by chromosome walking (Figure 7, Figure 8 and Figure 9). Bioinformatics analysis identified diverse functional elements in these promoters (Figure 7A, Figure 8A and Figure 9A): *DfHMGR1* contains RY-element (GA-inducible), Box 4 (light-responsive), and MYC (jasmonate signaling); *DfHMGR2* includes GC-motif (hypoxia-responsive) and STRE (stress-responsive); while *DfHMGR3* comprises STRE, LTR (low-temperature-responsive), and CGTCA-motif (MeJA-responsive) elements.

Transient transformation assays with five truncated fragments (full-length plus four truncations; Figure 7B, Figure 8B and Figure 9B) revealed distinct promoter activity patterns: *DfHMGR1* showed peak activity in the 649–863 bp region, *DfHMGR2* in 621–908 bp, and *DfHMGR3* in 0–362 bp (Figure 7D, Figure 8D and Figure 9D). GUS staining and luciferase reporter assays (Figure 7C, Figure 8C and Figure 9C) validated these active regions’ responsiveness: *DfHMGR1* responded specifically to PEG treatment (drought stress), and *DfHMGR2* to multiple stresses (drought, cold, MeJA, and salt), while *DfHMGR3* exhibited fragment-specific stress responses.

The identification of these stress-responsive elements led us to investigate the transcriptional regulatory network of *DfHMGR*s. Co-expression network analysis (Figure 10) elucidated *DfHMGR*s’ regulatory characteristics. The heatmap (Figure 10A) demonstrated consistent upregulation of *DfHMGR1–3* in trichomes (red blocks) but low expression in sporangia developmental stages and trichome-removed leaves (blue blocks). Weighted gene co-expression network analysis (WGCNA; Figure 10B) indicated an association between the MEmagenta module and *HMGR* (r = 0.56), with 21 candidate transcription factors identified in this module (Supplementary Table S4) potentially regulating *DfHMGR* expression. These findings provide crucial insights into the transcriptional regulation of *DfHMGR*s in *D. fragrans*.

## 4. Materials and Methods

### 4.1. Experimental Materials

#### 4.1.1. Plant Material

The plant materials used in this study included (1) one-year-old sporophytes of *Dryopteris fragrans* (spores were collected from wild populations in the Third Pool area of Wudalianchi National Nature Reserve, Heilongjiang Province, China) and (2) *Nicotiana tabacum* (Shanxi) and *Nicotiana benthamiana* plants cultivated in the laboratory greenhouse. All plants were maintained under standard growth conditions (22 °C, 50% relative humidity, 16 h light/8 h dark photoperiod).

#### 4.1.2. Culture Media

The study employed the following culture media systems: For microorganisms, LB medium (solid) (10 g/L tryptone, 5 g/L yeast extract, 10 g/L NaCl, 15 g/L agar, pH 6.8–7.0) and LB medium (liquid) (10 g/L tryptone, 5 g/L yeast extract, 10 g/L NaCl, pH 6.8–7.0). For *Nicotiana tabacum* (Shanxi) and *Nicotiana benthamiana*, seed germination medium M0 (1/8 MS + 30 g/L sucrose + 7 g/L agar, pH 5.8–6.0), leaf disk pre-culture/co-culture medium M1 (MS + 30 g/L sucrose + 7.5 g/L agar + 1.0 mg/L 6-BA + 0.1 mg/L NAA, pH 5.8–6.0), selection medium M2 (M1 + 50 mg/L cefotaxime + 50 mg/L kanamycin), and rooting medium M3 (1/2 MS + 30 g/L sucrose + 7 g/L agar, pH 5.8–6.0). For *Dryopteris fragrans*, Knop’s solid medium (0.25 g/L NaH_2_PO_4_ + 0.25 g/L KNO_3_ + 0.25 g/L MgSO_4_ + 1 g/L Ca(NO_3_)_2_·H_2_O + 7.5 g/L agar, pH 5.7–5.8) and gametophyte subculture medium (1/2 MS + 10 g/L sucrose + 7.5 g/L agar, pH 5.8–6.0) were used. All media were autoclaved at 121 °C for 15 min, while phytohormones and antibiotics were filter-sterilized through 0.22 μm membranes.

#### 4.1.3. Vectors and Strains

The eukaryotic expression vector pCAMBIA2300-35S-AD-nos, subcellular localization vector pCAMBIA2300-EGFP, prokaryotic expression vector pET28a(+), high-efficiency PCR product cloning vector pEASY-T18, and promoter activity analysis vectors pGreen II 0800-Luc and PBI121-GUS were maintained as plasmids in our laboratory. Competent cells of *Escherichia coli* DH5α, *Agrobacterium rubi* LBA4404, and prokaryotic expression strain BL21-Star™ (DE3) were purchased from Weidi Biotechnology (Shanghai, China).

#### 4.1.4. Major Reagents

Antibiotics including ampicillin (Amp), kanamycin (Kan), rifampicin (Rif), hygromycin (Hyg), and cefotaxime (Cef), along with plant growth regulators such as acetosyringone (As), 6-benzylaminopurine (6-BA), 2,4-dichlorophenoxyacetic acid (2,4-D), kinetin (KT), α-naphthaleneacetic acid (NAA), abscisic acid (ABA), methyl jasmonate (MeJA), ethephon (Eth), and salicylic acid (SA) were obtained from Solarbio Science & Technology (Beijing, China).

Molecular biology reagents including 2× Rapid Taq Master Mix, 2× Phanta Max Master Mix, HiScript II Q RT SuperMix for qPCR (+gDNA wiper), ChamQ Universal SYBR qPCR Master Mix, HiScript III 1st Strand cDNA Synthesis Kit (+gDNA wiper), and ClonExpress II One Step Cloning Kit were acquired from Vazyme Biotech (Nanjing, China). Agarose gel extraction kits and PCR product purification kits were provided by Sparkjade Biotechnology (Shandong, China). Specialized kits including the Plant Polysaccharide & Polyphenol RNA Extraction Kit (spin-column), plasmid DNA extraction kit, and Trelief Prestained Protein Ladder were sourced from Tsingke Biotechnology (Beijing, China). The One-Step Plant Polysaccharide & Polyphenol DNA Extraction Kit was obtained from Noble Biosciences (Beijing, China). Restriction enzymes and corresponding buffers were products of New England Biolabs (Ipswich, MA, USA).

Additional supplies including NuClean Plant Genomic DNA Kit, 6× Loading Buffer, 2K & 15K Marker, and TransZol Up were purchased from TransGen Biotech (Beijing, China). Murashige & Skoog (MS) Basal Medium with Vitamins was obtained from PhytoTechnology Laboratories (Lenexa, KS, USA). Conventional solvents including ethanol, isopropanol, glycerol, glacial acetic acid, and chloroform were supplied by Tianjin Fuyu Fine Chemical (Tianjin, China). Agarose, agar powder, tryptone, yeast extract powder, IPTG, and EDTA were purchased from Biotopped Science & Technology (Beijing, China). Polyethylene glycol 6000 (PEG6000), 2-(N-morpholino)ethanesulfonic acid (MES), and mannitol were Sigma-Aldrich products (Sigma Aldrich, St. Louis, MO, USA). Inorganic salts including NaH_2_PO_4_, KNO_3_, MgCl_2_, MgSO_4_, Ca(NO_3_)_2_·H_2_O, sodium chloride, and sucrose were obtained from Tianjin Tianli Chemical Reagent (Tianjin, China).

### 4.2. Bioinformatics Analysis

#### 4.2.1. Sequence Retrieval and Primary Characterization

The study commenced with retrieving the amino acid sequences of *A. thaliana* AtHMGR proteins from the NCBI database as references. Using TBtools-II (Toolbox for Biologists) v2.026 software [40], we screened the *D. fragrans* transcriptome database to identify DfHMGR protein sequences exhibiting high similarity to AtHMGR and determined their corresponding gene IDs. By comparing different transcriptome datasets of *D. fragrans*, we ultimately acquired the complete CDSs of three *DfHMGR* genes. Subsequently, we conducted in-depth analysis of DfHMGR protein sequences using multiple bioinformatics approaches: open reading frames were identified and amino acid sequence characteristics were analyzed using NCBI ORFfinder (https://www.ncbi.nlm.nih.gov/orffinder/, accessed on 16 September 2023) and key physicochemical parameters including molecular weight, theoretical isoelectric point, amino acid composition, and instability index were systematically evaluated via the ExPASy ProtParam online platform (https://web.expasy.org/protparam/, accessed on 27 September 2023). All analyses were performed using default parameter settings of each tool to ensure result reliability and comparability.

#### 4.2.2. Phylogenetic Reconstruction

Phylogenetic reconstruction was initiated by retrieving HMGR protein sequences homologous to DfHMGRs from diverse plant lineages using NCBI Blastp (https://blast.ncbi.nlm.nih.gov/Blast.cgi?PAGE = Proteins, accessed on 7 October 2023). The evolutionary tree was constructed with MEGA 11.0 software [41] employing the Neighbor-Joining algorithm (1000 bootstrap replicates). Based on the branching patterns, representative sequences closely related to DfHMGRs (including HMGRs from *A. nelumboides*, *C. richardii*, and *A. capillus-veneris*) were selected for multiple sequence alignment analysis performed with DNAMAN (Lynnon Corporation, San Ramon, CA, USA).

#### 4.2.3. Protein Structural Characterization and Subcellular Localization

For structural characterization, protein secondary structures were predicted using the NPS@ server (https://npsa-prabi.ibcp.fr/). Tertiary structure models were generated through homology modeling with SWISS-MODEL (https://swissmodel.expasy.org/), utilizing templates from the AlphaFold Protein Structure Database (AFDB). Subcellular localization predictions were performed with SoftBerry (http://www.softberry.com/, accessed on 20 October 2023).

#### 4.2.4. Promoter Cis-Element Analysis

The potential cis-acting elements in the promoter regions of *DfHMGR* genes were systematically analyzed using the PlantCARE database [42] (http://bioinformatics.psb.ugent.be/webtools/plantcare/html/, accessed on 25 October 2023) with default parameters. The analysis covered 2000 bp upstream of the transcription start sites and identified three major categories of functional elements.

#### 4.2.5. Co-Expression Network Construction

Gene expression analysis was performed using FPKM-normalized RNA-seq data from eight experimental conditions (three sporangia developmental stages, whole leaves, MeJA-treated, UV-treated, trichome-removed leaves, and trichomes). Hierarchical clustering was conducted with the pheatmap package (v1.0.12; https://cran.r-project.org/web/packages/pheatmap/, accessed on 15 November 2023) using Pearson correlation coefficients (range: −2 to 2) with Euclidean distance and the Ward.D2 linkage method. Statistical significance was assessed by *t*-test (α = 0.05).

Gene co-expression networks were constructed using the WGCNA package [43,44] (v1.72; https://cran.r-project.org/web/packages/WGCNA/, accessed on 15 November 2023) in R (v4.2.0) with a soft threshold power of 16. Module–trait relationships were analyzed by calculating Pearson correlations (range: −1 to 1) between module eigengenes and six MVA pathway genes (*AACT*, *HMGS*, *HMGR*, *MK*, *PMK*, and *MDC*), with *p* < 0.05 considered significant.

Candidate transcription factors were identified from co-expressed genes using (1) GFAP [45] for DNA-binding domain prediction (Pfam 35.0; E-value < 1 × 10^−5^); (2) PlantTFDB 5.0 [46] for family annotation; and (3) MEME Suite (v5.5.0) [47] for motif analysis (width: 6–12 bp, max iterations: 50). This yielded 21 candidate regulators with confirmed DNA-binding domains.

### 4.3. Experimental Procedures

#### 4.3.1. Cloning of the *DfHMGR*s

Total RNA was isolated from whole *D. fragrans* plants using the Plant Polysaccharide & Polyphenol RNA Extraction Kit (spin-column). RNA quality was assessed using an Implen NanoPhotometer^®^ P330 and 1% agarose gel electrophoresis. cDNA was synthesized from total RNA using the HiScript III 1st Strand cDNA Synthesis Kit (+gDNA wiper). The full-length CDSs of *DfHMGR1*, *DfHMGR2*, and *DfHMGR3* were analyzed to identify ORFs using NCBI BLAST (https://blast.ncbi.nlm.nih.gov, accessed on 20 November 2023). The ORFs were compared with other plant *HMGR* genes to verify the presence of start and stop codons and assess length conservation. Gene-specific primers (Table S5) were designed flanking the ORFs.

PCR amplification was performed using 2× Phanta Max Master Mix. After gel electrophoresis verification, PCR products were purified using Agarose Gel Extraction Kits and PCR Product Purification Kits. Purified products were ligated into the pEASY-T18 vector and transformed into *E. coli* DH5α-competent cells via heat shock. Positive clones were verified by colony PCR and Sanger sequencing (Ruibo Biotech, Beijing, China). Sequence analysis and alignment were conducted using SnapGene^®^ 6.0.2 software (http://www.snapgene.com) to confirm successful ligation. Verified clones were cultured in 10 mL LB liquid medium containing 100 mg/L Amp at 37 °C with 200 rpm shaking for 12–16 h. For storage, 800 μL of bacterial culture was mixed 1:1 with 30% glycerol, flash-frozen in liquid nitrogen, and stored at −20 °C. The remaining culture was used for plasmid extraction with a Plasmid DNA Extraction Kit, and the purified plasmids were stored at −20 °C for subsequent experiments.

#### 4.3.2. DfHMGR Protein Expression

The pET-28a(+) vector was linearized by HindIII restriction endonuclease (New England Biolabs) digestion at 37 °C for 12 h, followed by gel extraction purification. Homology arm primers (Table S5) were designed to amplify *DfHMGR* fragments. The purified PCR products were recombined with the linearized vector using the ClonExpress II One Step Cloning Kit (Vazyme, Nanjing, China). Recombinant plasmids were transformed into *E. coli* DH5α-competent cells (Weidi Biotechnology, Shanghai, China), with positive clones verified by sequencing.

For protein expression, verified plasmids were transformed into BL21-Star™ (DE3) cells (Weidi Biotechnology). Cultures were grown in LB medium (50 mg/L kanamycin) at 37 °C until OD_600_ reached 0.6. IPTG was added at three concentrations (0.5, 1.0, 1.5 mM), with each concentration group induced for 1, 2, 4, and 8 h (37 °C, 200 rpm) to determine optimal conditions.

#### 4.3.3. Identification of Subcellular Localization of DfHMGRs

The pCAMBIA2300-EGFP was digested with BamHI-HF and purified by gel extraction. Primers for the DfHMGR subcellular localization vector homology arms were designed (Supplementary Table S5). Positive *A. rubi* was cultured in LB medium (50 mg/L Kan, 20 mg/L Rif) until OD_600_ reached 1.0–1.2. After centrifugation, cells were resuspended to OD_600_ 0.6–0.8 in infiltration buffer (10 mM MgCl_2_, 10 mM MES, 150 μM acetosyringone) and incubated at room temperature for 4–6 h in the dark.

Four-week-old N. benthamiana plants were pre-watered. Bacterial suspensions were injected into abaxial leaf surfaces using sterile 1 mL syringes. Plants were incubated under dark (24 h), low-light (24 h), and normal-light (48 h) conditions. Fluorescence was observed by confocal microscopy (excitation: 450–490 nm for blue, 520–550 nm for green) after 48 h.

#### 4.3.4. qPT-PCR Analysis of *DfHMGR* Expression Patterns

Ten uniform annual sporophytes of *D. fragrans* were divided into eight treatment groups: (1) hormonal treatments including 100 μM salicylic acid (SA), 10 μM abscisic acid (ABA), 1.2 mM methyl jasmonate (MeJA), and 500 μM ethylene (ETH), with distilled water as control; (2) abiotic stress treatments comprising 20% PEG6000 irrigation, 200 mM NaCl irrigation, 42 °C heat stress, and 4 °C cold stress, using 24 °C as control. Tissue samples were collected at 0, 0.5, 1, 3, 6, 12, 24, and 48 h post-treatment. Total RNA was extracted using the Plant Polysaccharide & Polyphenol RNA Extraction Kit (Tsingke Biotechnology, Beijing, China) and reverse-transcribed with HiScript III 1st Strand cDNA Synthesis Kit (+gDNA wiper; Vazyme, Nanjing, China). qRT-PCR was performed on a LineGene 9620 Real-Time PCR System (Bioer Technology, Hangzhou, China) using ChamQ Universal SYBR qPCR Master Mix (Vazyme) under the following conditions: 95 °C for 30 s, then 40 cycles of 95 °C for 10 s and 60 °C for 30 s. The relative expression levels were calculated by the 2^−ΔΔCt^ method using 18S rRNA as the internal control with three biological replicates. Gene-specific primers (Table S5) were designed with Primer Premier 6.00 and synthesized by Ruibo Biotech (Harbin, China).

#### 4.3.5. Constructing *DfHMGR*s Overexpressing *N. tabacum*

The *DfHMGR*-overexpressing transgenic *N. tabacum* plants were generated through *Agrobacterium*-mediated transformation. Based on the *DfHMGR* gene sequences and pCAMBIA2300-35S-AD-nos vector map, the target genes were inserted via EcoRI digestion and homologous recombination to construct recombinant plasmids (2300-35S-AD-*DfHMGR1/2/3*-nos). These plasmids were sequentially transformed into *E. coli* DH5α and *A. rubi* LBA4404. Bacterial suspensions (OD_600_ = 0.6–0.8) were used to infect pre-cultured *N. tabacum* leaf disks. After 3 days of co-cultivation, transformants were selected on kanamycin-containing medium (50 mg/L) and verified by PCR amplification (857 bp target fragment) using vector-specific primers (Supplementary Table S5).

For phenotypic characterization, homozygous T3 transgenic lines were subjected to germination assays under different NaCl concentrations (0, 50, and 100 mM) to measure germination rates and root lengths under stress conditions. Germination assays were conducted with three replicate plates per group (WT and *DfHMGR1–3* transgenic lines), with 30 seeds per plate. Physiological analyses including SOD, POD, CAT, and MDA activities were performed following high-temperature (42 °C for 4 h) and drought (2 weeks) treatments. Three biological replicates were performed for each experiment. Statistical analysis was conducted using one-way ANOVA with Tukey’s test (*p* < 0.05) in DPS 12.01 software [48] (http://www.dpsw.cn), with different lowercase letters indicating significant differences.

#### 4.3.6. Cloning and Truncation of the *DfHMGR* Promoter

Genomic DNA was extracted from *D. fragrans* leaves using the One-Step Plant Polysaccharide & Polyphenol DNA Extraction Kit (Noble Biosciences, Beijing, China) according to the manufacturer’s instructions. DNA quality was verified by microspectrophotometric quantification and 1% agarose gel electrophoresis. To obtain the 5′ upstream sequences, we performed chromosome walking through FPNI-PCR (Fusion Primer and Nested Integrated PCR) using a combination of published FP1–9 primers [49] and our custom-designed FP10–16 primers with *DfHMGR*-SP1 as the reverse primer (all primer sequences in Supplementary Table S5). The three-round PCR amplification involved successive reactions with a genomic DNA template and random primers (FP1–FP16 plus *DfHMGR*-SP1), first-round PCR products with FSP1 and *DfHMGR*-SP2, and 50-fold-diluted second-round products with FSP2 and *DfHMGR*-SP3. PCR products showing the expected 100–200 bp size reduction between rounds were sequenced, and promoter regions were confirmed by 3′-5′ sequence alignment. For *DfHMGR1* and *DfHMGR2* requiring extended walking, full-length promoters were amplified using gene-specific primers Pro*DfHMGR1*-full-F/*DfHMGR1*-SP6 and Pro*DfHMGR2*-full-F/*DfHMGR2*-SP9 (Table S5).

Through comprehensive analysis of cis-acting element distribution in the promoters, we designed truncation primers to strategically preserve functional domains while avoiding critical regulatory elements (Supplementary Table S5). The primer design ensured that identical or functionally similar binding sites were maintained within the same truncated fragments, while distinct functional elements were separated. Using the full-length promoter sequences as templates, PCR amplification with Pro*DfHMGR*-T1-F, Pro*DfHMGR*-T2-F, Pro*DfHMGR*-T3-F, Pro*DfHMGR*-T4-F and Pro*DfHMGR*-R primers successfully generated four truncated promoter fragments (T1–T4) for functional characterization.

#### 4.3.7. Identification of the Transcriptional Activity of the *DfHMGR* Promoter

We constructed Pro*DfHMGR*-full/T1/T2/T3/T4-EGFP::GUS and pGreenII-0800-Pro*DfHMGR*-full/T1/T2/T3/T4::luc vectors, followed by BamHI single digestion according to the vector map. The digested vectors were recombined with homologous arm-attached promoter fragments and transformed into *E. coli* DH5α-competent cells. These plasmids were introduced into *A. rubi* LBA4404 via electroporation. Verified positive colonies were used for transient transformation of *N. benthamiana* leaves.

For GUS staining, infiltrated *N. benthamiana* leaves were vacuum-infiltrated with staining solution for 30 min, then incubated at 37 °C for 12–16 h. After removing the staining solution, tissues were decolorized with 95% ethanol (changed every 12 h at room temperature) until complete chlorophyll removal, then preserved in 95% ethanol.

## 5. Conclusions

This study investigated three *HMGR* genes (*DfHMGR1–3*) in *Dryopteris fragrans*, revealing their roles in abiotic stress responses. We demonstrated that all three DfHMGR proteins localize to the endoplasmic reticulum. These genes exhibited distinct expression patterns under various stresses, with particularly strong induction by ABA (up to 8.45-fold) and salt stress (up to 9.48-fold). Transgenic *N. tabacum* plants overexpressing *DfHMGR*s showed enhanced tolerance to multiple stresses, including improved germination rates under salt stress and better leaf integrity during drought. Promoter analysis identified functional regulatory regions controlling *DfHMGR* expression. Co-expression network analysis uncovered 21 potential transcriptional regulators that may control *DfHMGR* expression. These results establish *DfHMGR*s as potential candidate genes for plant stress resistance research based on heterologous expression evidence.

## Figures and Tables

**Table 1:** Basic characteristics of DfHMGRs.

	Total Length of Coding Sequence (CDS)	Number of Coded Amino Acids	Relative Molecular Mass of Proteins	Isoelectric Point (pI)	Aliphatic Index	Grand Average of Hydropathicity (GRAVY)
HMGR1	1722 bp	573	61.2 kDa	7.19	33.26	0.05
HMGR2	1713 bp	570	60.9 kDa	6.22	43.16	0.179
HMGR3	1737 bp	578	61.7 kDa	8.05	40.25	0.136

## Data Availability

The original contributions presented in this study are included in the article/Supplementary Materials. Further inquiries can be directed to the corresponding authors.

## Supplementary Materials

The following supporting information can be downloaded at https://www.mdpi.com/article/10.3390/plants14142190/s1, Figure S1: *Dryopteris fragrans* collected from Wudalianchi, Heilongjiang Province, China; Figure S2: Secondary structure prediction of DfHMGRs; Figure S3: Tertiary structure prediction of DfHMGRs; Figure S4: Results of multi-sequence alignment of DfHMGRs; Figure S5: Inducible expression of DfHMGRs; Figure S6. Generation of transgenic *N. tabacum* plants overexpressing *DfHMGR* genes; Figure S7. PCR identification of transgenic *N. tabacum* plants overexpressing *DfHMGR*s; Table S1: Taxonomic classification and protein nomenclature of HMGR sequences used in phylogenetic analysis; Table S2: CDSs of the *DfHMGR* gene; Table S3: Amino acid sequence of DfHMGR protein; Table S4: Basic properties of transcription factor candidates; Table S5: Primer sequences.
