# Supplementary material for: Functional Identification and Transcriptional Activity Analysis of Dryopteris fragrans HMGR Gene
Source: Plants (Basel). 2025 Jul 15;14(14):2190. doi: 10.3390/plants14142190 (PMC12301065; doi:10.3390/plants14142190)
Supplement: Supplementary file 1 [file plants-14-02190-s001.zip › plants-3582467-supplementary.pdf]

Table S1. Taxonomic classification and protein nomenclature of HMGR sequences used in phylogenetic analysis

Protein ID	Species name	Family name	Genus name	Plant group
DfHMGR	*Dryopteris fragrans* (L.) Schott	Dryopteridaceae	*Dryopteris*	Pteridophyta
CrHMGR	*Ceratopteris richardii* Brongn.	Pteridaceae	*Ceratopteris*	Pteridophyta
AnHMGR	*Adiantum nelumboides* X. C. Zhang	Pteridaceae	*Adiantum*	Pteridophyta
AcHMGR	*Adiantum capillus-veneris* L.	Pteridaceae	*Adiantum*	Pteridophyta
SmoHMGR	*Selaginella moellendorffii* Hieron.	Selaginellaceae	*Selaginella*	Pteridophyta
DcHMGR	*Diphasiastrum complanatum* (L.) Holub	Lycopodiaceae	*Diphasiastrum*	Pteridophyta
PpHMGR	*Physcomitrella patens* (Hedw.) Bruch & Schimp.	Funariaceae	*Physcomitrella*	Bryophyta
SfHMGR	*Sphagnum fallax* (H. Klinggr.) H. Klinggr.	Sphagnaceae	*Sphagnum*	Bryophyta
SmaHMGR	*Sphagnum magellanicum* Brid.	Sphagnaceae	*Sphagnum*	Bryophyta
MpoHMGR	*Marchantia polymorpha* L.	Marchantiaceae	*Marchantia*	Bryophyta
MpaHMGR	*Marchantia paleacea* Bertol.	Marchantiaceae	*Marchantia*	Bryophyta
GbHMGR	*Ginkgo biloba* L.	Ginkgoaceae	*Ginkgo*	Gymnospermae
TmHMGR	*Taxus* × *media* Rehder	Taxaceae	*Taxus*	Gymnospermae
CjHMGR	*Cryptomeria japonica* (Thunb. ex L. f.) D. Don	Cupressaceae	*Cryptomeria*	Gymnospermae
NaHMGR	*Nicotiana attenuata* Torr. ex S.Watson	Solanaceae	*Nicotiana*	Angiospermae
NtHMGR	*Nicotiana tabacum* L.	Solanaceae	*Nicotiana*	Angiospermae
NsHMGR	*Nicotiana sylvestris* Speg.	Solanaceae	*Nicotiana*	Angiospermae
NbHMGR	*Nicotiana benthamiana* Domin	Solanaceae	*Nicotiana*	Angiospermae
PaHMGR	*Prosopis alba* Griseb.	Fabaceae	*Prosopis*	Angiospermae
RcHMGR	*Ricinus communis* L.	Euphorbiaceae	*Ricinus*	Angiospermae
PvHMGR	*Pistacia vera* L.	Anacardiaceae	*Pistacia*	Angiospermae
ZjHMGR	*Ziziphus jujuba* Mill.	Rhamnaceae	*Ziziphus*	Angiospermae

Table S2. CDS sequence of the *DfHMGRs* gene

Gene ID	CDS sequence
HMGR1	ATGGGATTGCATTATTATGATCGTAGTTCCTTTGATGAGAAGCATTCAAGGCGCAACAAGAACTCCAACTCCAAGCTGCTT<br>GCTTCAGATGCCCTTCCACTTCCAGTGTGGCTCACAAACAGGATATTCATTGTGCTATTTTGTGCTGCATCGTACCATCTCT<br>TGAGAAGATGGGGGGAGAAAAAAGTCAGGACACCCACTCCCCTTTATGTCCTGAGCTTTGGAGACATGGTAGCATTTGTA<br>GGCCTCATTGCTTCTCTTATTTATCTCTTGGGTTTTTTTGGGATCGATTATGTTCAAAACTTCATTGGCAAGGGTTCTGATG<br>GGCACTGGGATTTTGTTGATGAAACGGAAGGGCTTGTTCGAGGTTGTCGTAGCGGGAAGGCAACAGAAGGCGCTCTCCCA<br>TCGGTACCACCAGAAACGAATCTCGATGGTCAGAATTTGAAGGAGAGCAACGATGAGGACATTGCGTGGGCAGTTAGTC<br>GTGGATCTGTAGCCTCGCATACATTGGAAGGTAGTGTGGGAGATGCACAACGGGCAGTATCAATAAGGAAGAGGTCAGT<br>AGAGCTTATGACTGGCAGGTCATTGGAAAAGCTTTCACTTGGGGGGTTTGACTACTCAGCTGTGGTTGGGCAGTGCTGCG<br>AAATGGTCATCGGGCATGTACAATTACCCGTTGGTGTTGCAGGGCCTCTTATGCTCGATGGGTTTGATTGTATGGTACCTA<br>TGGCTACCACAGAGGGCTGCCTTGTCGCAAGCACCAATCGTGGGTGCAAGGCTATACATCTGTCTGGAGGAGCAACAAGC<br>ATTCTCCTCAGAGACGGGATGACAAGGGCACCTGTGGTCCGCTTTGACAAAGCTCATCGTGCTGCAGAGCTCAAGTTCTA<br>CGTAGAGGACACCAACAATTTTGACACGCTAAGTGTTATTTTTAACAGGACCAGTAGATTTGCACGGTTACAGTATATTCA<br>ATGTGCTATGGCGGGTCGCAATTTGTATCTCCGTTTTAGCTGCTCGACAGGAGATGCAATGGGGATGAACATGGTCTCCAA<br>AGGGGTGCAAAATGTCCTAGAATACCTCCAGGACGTGTTTCCAGATATGGAGATTTTAAGTCTTTCAGGCAATTTTTGTGC<br>GGATAAAAAGGCAACGGCTGTGAATTGGATTAATGGGCGCGGAAAGTCGGTTGTATGTGAGGCGGTTATCAGCGGTGAC<br>GTAGTGGCGAAGGTGCTCAAGACATCGGTGTCTAGTTTGGTGGAGCTCAATTCAGTGAAGAATTTATCGGGCTCGGCTAT<br>TGCGGGTGCCCTGGGAGGATTCAATGCGCACGCTGCCAACATTGTGTCTGCTGTTTTCATTGCAACTGGTCAAGATCCTGC<br>CCAAAATGTGGAGAGTTCTCATTGTATTACTATGATGGAGCCTACAAATGGTGGACTTGACCTACATGTATCTGTCACAAT<br>GCCATCTTTGGAGGTGGGGACGATTGGAGGTGGAACACAGCTTGAATCGCAATCTGCATGCTTGAACCTTTTGGGAGTGA<br>AAGGAGCTAACGCCGACTTACCGGGTGCTAATGCACAAAAGCTTGCAAGGGTGGTTGCTGGGGCTGTTTTGGCTGGAGAG<br>TTGTCACTCATGTCAGCTTTAGCTGCCGGCCATCTCGTAAAGAGTCATATGAGATACAATCGGTCGAGCAAGAATCTGGC<br>CTCCGAAGGCCAACACAAAGAGTGTTCGACTTGA
HMGR2	ATGGGTGCAGACTTTCTTTCTGGGGTCCCAATCCAGGAGAAGAGTCCGTCTGACAAGAAGAATGTGAAACTCCTTGCCTC<br>AGATGCGCTACCATTACCAGTCTGGTTGACAAACAGGGTCTTCTTTGTTCTATTTTTTGCAGCAACTTATTATCTCATGAGA<br>AGATGGGGAGAGAAGAAGGGCCGAGTTCCTACGCCCCTCCATATTTTGAGTTTTGGAGATATTGTTGCTTTGGTTGTCGTT<br>GTTGCCTCTTTCATATATCTATTGGGCTTCTTTGGAATCGATTATGTACAACAAGCAATCAGATCAGCAGATGGTCAATGG<br>GACTTCAATGAGGAAACAGAAGTTCTTGTTCAAGATAAGGCTTGCCTTCCTACTCCTCAGGTCCCTAGGCCAATTGTACAA<br>CTCAAGGGCCAGGATATACGTGACAACACGGATGAAGACATTGCCTGTGCAGTTAGTTGTGGTTCTGTTCCATCTTACTCT<br>CTGGAAGATAGCTTAGGAGATGCTGAAAGGGGGGCTTCTATCAGAAAGCGAGCAATTGAGATTATGACTGGAAGGTCCCT<br>TGAGGGTCTCCCCCTTGGAGGTTTGAATTACTCAGCTGTTTTGGGACAATGCTGTGAAATGGTGATTGGACACGTGCAAAT<br>TCCGGTTGGAGTTGCGGGACCCCTTCTTATGGACGGATATGAGTGCATGGTGCCCATGGCAACAACTGAAGGCTGTCTTGT<br>GGCAAGCACCAACCGAGGGTGCAAGGCTATACACATGTCGGGTGGCGCAACAACTGTCCTCCTTAGGGATGGCATGACA<br>AGGGCTCCAGTAGTAAGATTTGAGAGTGCTCAGCGTGCTTCTGAGTTGAAATTCTACATTGAGAATGCTGAAAACTTTGA<br>GACAATTTGCGTCATGTTCAACAGGTCAAGCAGGTTTGCCCGGTTACAGTATATCCAATGTGCCCTGGCTGGACGCAATCT<br>GTACTTGCGCTTTAGCTGCTCAACGGGAGATGCTATGGGGATGAACATGGTGTCAAAGGGTGTCCAGAATGTGCTAGAGT<br>ATTTGCAGCACGTGTTCCCTGATATGGAAGTTATGAGCTTGTCTGGCAACTTCTGTGCAGATAAGAAGGCCACTGCGGTCA<br>ATTGGATAAATGGACGTGGCAAGTCTGTGGTTTGTGATGCTGTGATCAAAGGGGAGGTGGTTGCCAAGGTCTTGAAAACA<br>TCAGTCTCGGCATTGGTAGAACTTAATATCATAAAAAATCTGACGGGATCTGCTGTTGCTGGTGCATTGGGTGGGTTCAAT<br>GCTCATGCATCCAACATAGTGTCTGCCGTTTTCCTTGCCACCGGCCAAGATCCGGCACAGAATATTGAAAGTTCTAGCTGT<br>ATCACATTAATGGAAGCAGTCAATGATGGACAGGATCTCTATGTGTCCGTAACGATGCCATCTATTGAGGTGGGGACGGT<br>TGGAGGTGGAACACAATTACCTTCTCAGTCTGCATGTTTGAATCTGCTGGGAGTTAAAGGCGCAAACTTAGATTTACCTGG<br>TTCAAATGCACAGAAGCTTGCAAGAGTGGTTGCTGGATCAGTTTTGGCTGGAGAGTTGTCCTTAATGTCAGCTCTTGCTGC<br>AGGGCATCTCGTCAAGAGTCACATGAAGTACAATCGGTCGACCAAGAACATATCCGTCGAATCAGTAACCTCGGACGTAC<br>AAAACGCAAAATGTGCCTAG
HMGR3	ATGGATCCTTTCCCCTGCGACGAGCGCGCCTGCATTGGCTTCTCCGGTAAGCAAAATCAACATAGCCAAAACAGCATGAA<br>ACGCGTCAAGAGCTCCAAGCTTTTGCTTGCTTCTGATGCCCTCCCTTTGCCTGTTGGCCTCACTAACAAACTCTTCCTTTTC<br>CTCTTCTTTGCCGCATCGTACTACCTGCTCAGACGATGGGGAGAGAAGAAAAAAGCTAACATTCCCCTTCATGACCTCAG<br>CTTCACAGAAATCGTAGCCGTGCTCACGCAGATTGCGTCCATTATTTACCTATGGGGCTTTTTCGGTATAGTGTATGTTCA<br>GAACTGCATCAGCAAAGGAGAAGATGAGAGAGATAGCATGCTAGGGTCTGCCCTGACAAGGTCACCTGTGCCGCCTTCG<br>CACCCTCTTGCAGCACCTCGGCCCCCATTACTGCTTAAAGGCCAAGATTTGAAGGAAAACAGGGATGAAGACATTGCATG<br>TGCAGTTAGTGAGGGTGAAGTGCCCTCTTATTCTTTAGAAAAGGACCTCGGAGATTGCACTAGGGCAGCCTCTGTGAGGA<br>AACGCGCAGTGGAGATGATGACAGGAAGGTCTTTAGAAGCCCTCCCATTGGACAATTTCAACTATGCGTCTGTTCTTGGG<br>CAATGCTGCGAGATGGTGATTGGGTTCGTGCAGATCCCTGTTGGTGTGGCTGGCCCCCTCCTCCTCGATGGCTTCGAGTAC<br>ATGGTCCCCATGGCCACCACTGAGGGCTGCCTTGTTGCAAGCACGAATCGTGGATGCAAGGCCATTCACCTCTCAGGTGG<br>TGCATCCAGCATTCTTCTCCAAGATGGCATGACACGGGCACCCGTCGTCCGCTTCACATCAGCAGTGCGTGCTTCAGAGTT<br>GAAGTTTTTTGTGGAGGCAGGGGAGAATTTGGAGATGCTTTCCTTGGTGTTTAATAAGTCAAGTAGGTTTGCAAGGCTGCA<br>AAGCATCCAGTGTTCGTTGGCAGGCCGCAACTTGTATATGCGCTTCAAATGCTCGACAGGCGATGCAATGGGGATGAACA<br>TGGTATCAAAAGGGGTTCAAAATGTTCTCGATTATCTGCAGCATGTCTTTCCTGATATGGAAGTTGTCAGCCTTTCGGGCA<br>ACTTCTGTGCTGACAAAAAGGCTACTGCTGTCAATTGGATTCAAGGACGCGGCAAGTCTGTCGTATGTGAGGCTGTTATCA<br>AAGAGGAGGTTGTGGCCAAAGTGTTAAAGACATCCGTGGCAGCACTTGTGGAACTAAACATGATAAAAAATCTTACGGG<br>CTCTGCAATAGCTGGTGCTCTTGGTGGTTTCAATGCACATGCAGCAAACATTGTTTCAGCTGTCTTTCTTGCGACAGGCCA<br>AGACCCAGCTCAAAATGTAGAGAGCTCTCAATGCCTTACAATGATGGAGACAACCAATGGTGGCCGTGACTTACATATAT<br>CTGTGACTATGCCTTCTGTAGAGGTGGGTACAATTGGGGGTGGGACACAGTTGGCATCACAATCAGCATGCTTGAATATG<br>CTAGGAGTAAAAGGTGCAAATGTAGATTCACCAGGAGCTAATGCGCAATGTTTAGCAAGAATCATAGCAGGATCGGTGCT<br>CGCCGGAGAATTATCCCTCATGTCTGCTCTTGCTGCCGGCCACCTAGTAAAGAGCCACATGAAATATAACCGATCTAGTA<br>AAAACATCTGTCTAGAAGCTGTTGCTGATGGCTCACAAGGAAAGATGTAA

Table S3. Amino acid sequence of DfHMGRs protein

Protein ID	Amino acid sequence
HMGR1	MGLHYYDRSSFDEKHSRRNKNSNSKLLASDALPLPVWLTNRIFIVLFCAASYHLLRRWGEKKVRTPTPLYVLSFGDMVAFVGLIASLIYLLGFFGIDYVQNFIGKGSDGHWDFVDETEGLVRGCRSGKATEGALPSVPPETNLDGQNLKESNDEDIAWAVSRGSVASHTLEGSVGDAQRAVSIRKRSVELMTGRSLEKLSLGGFDYSAVVGQCCEMVIGHVQLPVGVAGPLMLDGFDCMVPMATTEGCLVASTNRGCKAIHLSGGATSILLRDGMTRAPVVRFDKAHRAAELKFYVEDTNNFDTLSVIFNRTSRFARLQYIQCAMAGRNLYLRFSCSTGDAMGMNMVSKGVQNVLEYLQDVFPDMEILSLSGNFCADKKATAVNWINGRGKSVVCEAVISGDVVAKVLKTSVSSLVELNSVKNLSGSAIAGALGGFNAHAANIVSAVFIATGQDPAQNVESSHCITMMEPTNGGLDLHVSVTMPSLEVGTIGGGTQLESQSACLNLLGVKGANADLPGANAQKLARVVAGAVLAGELSLMSALAAGHLVKSHMRYNRSSKNLASEGQHKECST
HMGR2	MGADFLSGVPIQEKSPSDKKNVKLLASDALPLPVWLTNRVFFVLFFAATYYLMRRWGEKKGRVPTPLHILSFGDIVALVVVVASFIYLLGFFGIDYVQQAIRSADGQWDFNEETEVLVQDKACLPTPQVPRPIVQLKGQDIRDNTDEDIACAVSCGSVPSYSLEDSLGDAERGASIRKRAIEIMTGRSLEGLPLGGLNYSAVLGQCCEMVIGHVQIPVGVAGPLLMDGYECMVPMATTEGCLVASTNRGCKAIHMSGGATTVLLRDGMTRAPVVRFESAQRASELKFYIENAENFETICVMFNRSSRFARLQYIQCALAGRNLYLRFSCSTGDAMGMNMVSKGVQNVLEYLQHVFPDMEVMSLSGNFCADKKATAVNWINGRGKSVVCDAVIKGEVVAKVLKTSVSALVELNIIKNLTGSAVAGALGGFNAHASNIVSAVFLATGQDPAQNIESSSCITLMEAVNDGQDLYVSVTMPSIEVGTVGGGTQLPSQSACLNLLGVKGANLDLPGSNAQKLARVVAGSVLAGELSLMSALAAGHLVKSHMKYNRSTKNISVESVTSDVQNAKCA
HMGR3	MDPFPCDERACIGFSGKQNQHSQNSMKRVKSSKLLLASDALPLPVGLTNKLFLFLFFAASYYLLRRWGEKKKANIPLHDLSFTEIVAVLTQIASIIYLWGFFGIVYVQNCISKGEDERDSMLGSALTRSPVPPSHPLAAPRPPLLLKGQDLKENRDEDIACAVSEGEVPSYSLEKDLGDCTRAASVRKRAVEMMTGRSLEALPLDNFNYASVLGQCCEMVIGFVQIPVGVAGPLLLDGFEYMVPMATTEGCLVASTNRGCKAIHLSGGASSILLQDGMTRAPVVRFTSAVRASELKFFVEAGENLEMLSLVFNKSSRFARLQSIQCSLAGRNLYMRFKCSTGDAMGMNMVSKGVQNVLDYLQHVFPDMEVVSLSGNFCADKKATAVNWIQGRGKSVVCEAVIKEEVVAKVLKTSVAALVELNMIKNLTGSAIAGALGGFNAHAANIVSAVFLATGQDPAQNVESSQCLTMMETTNGGRDLHISVTMPSVEVGTIGGGTQLASQSACLNMLGVKGANVDSPGANAQCLARIIAGSVLAGELSLMSALAAGHLVKSHMKYNRSSKNICLEAVADGSQGKM

Table S4. Basic properties of transcription factor candidates

Gene ID	CDS Length (bp)	Protein Length (aa)	Molecular weight (Da)	pI	Predicting subcellular localization	Transcription Factor Subfamily
evm.model.LG40.463	474	157	17850.12	6.61	Nucleus.Peroxisome.	Myb DNA-binding domain 4
evm.model.LG37.486	498	165	18799.96	5.27	Chloroplast.Nucleus.	GRAS domain
evm.model.LG21.134	993	330	36777.01	9.13	Nucleus.	C2H2 zinc finger domain
evm.model.LG15.842	1752	583	62831.99	7.58	Nucleus.	No apical meristem (NAM) domain
evm.model.LG36.669	975	324	35766.67	6.76	Nucleus.	ZF-HD dimer domain
evm.model.LG10.782	567	188	21827.95	9.96	Nucleus.	Myb DNA-binding domain
evm.model.LG05.937	1413	470	48674.54	6.19	Nucleus.	AP2 domain
evm.model.LG31.839	456	151	16632.3	10.18	Nucleus.	Helix-loop-helix (HLH) domain
evm.model.LG22.642	1524	507	55121.13	6.46	Nucleus.	Lateral organ boundaries (LOB) domain
evm.model.LG08.845	1398	465	49246.5	5.87	Nucleus.	CCCH zinc finger domain
evm.model.LG33.587	732	243	26582.09	5.4	Nucleus.	bZIP domain
evm.model.LG04.1218	1473	490	53085.53	5.41	Nucleus.	AP2 domain
evm.model.LG24.556	519	172	19765.46	8.23	Nucleus.	Myb DNA-binding domain 4
evm.model.LG04.262	540	179	19054.41	5.91	Nucleus.	Lateral organ boundaries (LOB) domain
evm.model.LG11.495	972	323	36794.29	5.82	Nucleus.	Homeodomain
evm.model.LG22.290	3102	1033	114156.21	6.52	Nucleus.	PHD finger domain
evm.model.LG15.289	1122	373	41637.41	5.41	Nucleus.	Myb DNA-binding domain 4
evm.model.LG22.782	717	238	27224.65	8.23	Nucleus.	Myb DNA-binding domain 4
evm.model.LG08.251	2868	955	103434.5	6.12	Nucleus.	Homeodomain
evm.model.LG02.1106	2322	773	86041.84	5.66	Nucleus.	CCCH zinc finger domain
evm.model.LG22.607	999	332	37439.91	6.62	Nucleus.	AP2 domain

Table S5. Primer sequences

Primer	Sequence (5'→3')	Function
DfHMGR1-F	ATGGGATTGCATTATTATGATCGTAGTTC	*DfHMGRs* gene cloning
DfHMGR1-R	TCAAGTCGAACACTCTTTGTGTTG	
DfHMGR2-F	ATGGGTGCAGACTTTCTTTCTG	
DfHMGR2-R	CTAGGCACATTTTGCGTTTTGTAC	
DfHMGR3-F	ATGGATCCTTTCCCCTGCG	
DfHMGR3-R	TTACATCTTTCCTTGTGAGCCATCAG	
pET28a（+）-*DfHMGR1* Homologous arm-F	CAAATGGGTCGCGGATCCATGGGATTGCATTATTATGATCGT	Prokaryotic (protein) expression
pET28a（+）-*DfHMGR1* Homologous arm-R	GGAGCTCGAATTCGGACCAGTCGAACACTCTTTGTGTTG	
pET28a（+）-*DfHMGR2* Homologous arm-F	CAAATGGGTCGCGGATCCATGGGTGCAGACTTTCTTTCT	
pET28a（+）-*DfHMGR2* Homologous arm-R	GGAGCTCGAATTCGGACCGGCACATTTTGCGTTTTGTAC	
pET28a（+）-*DfHMGR3* Homologous arm-F	CAAATGGGTCGCGGATCCATGGATCCTTTCCCCTGC	
pET28a（+）-*DfHMGR3* Homologous arm-R	GGAGCTCGAATTCGGACCCATCTTTCCTTGTGAGCCATC	
DfHMGR1-EGFP-Homologous arm-F	CTCGGTACCCGGGGATCCATGGGATTGCATTATTATGATCG	Subcellular localisation
DfHMGR1-EGFP-Homologous arm-R	GGTGTCGACTCTAGAGGATCCAGTCGAACACTCTTTGTGT	
DfHMGR2-EGFP-Homologous arm-F	CTCGGTACCCGGGGATCCATGGGTGCAGACTTTCT	
DfHMGR2-EGFP-Homologous arm-R	GGTGTCGACTCTAGAGGATCCGGCACATTTTGCGTTTTGTA	
DfHMGR3-EGFP-Homologous arm-F	CTCGGTACCCGGGGATCCATCCATGGATCCTTTCCCCTG	
DfHMGR3-EGFP-Homologous arm-R	GGTGTCGACTCTAGAGGATCCCATCTTTCCTTGTGAGCCA	
18S-YG-F	GCTTTCGCAGTAGTTCGTCTTTC	qRT-PCR
18S-YG-R	TGGTCCTATTATGTTGGTCTTCGG	
DfHMGR1-YG-F	GGGTTCTGATGGGCACTGGGAT	
DfHMGR1-YG-R	TGTCCTCATCGTTGCTCTCCTTCA	
DfHMGR2-YG-F	GCAATCAGATCAGCAGATGGTCAA	
DfHMGR2-YG-R	CAGGCAATGTCTTCATCCGTGTT	
DfHMGR3-YG-F	GATGCCCTCCCTTTGCCTGTTG	
DfHMGR3-YG-R	GTGAGCACGGCTACGATTTCTGT	
HMGR1-2300-Homologous arm-F	GCCATGGAGGCCAGTGAATTCATGGGATTGCATTATTATGATCG	Overexpression vectors
HMGR1-2300-Homologous arm-R	CCCACCCGGGTGGAATTCTCAAGTCGAACACTCTTTGT	
HMGR2-2300-Homologous arm-F	GCCATGGAGGCCAGTGAATTCATGGGTGCAGACTTTCT	
HMGR2-2300-Homologous arm-R	CCCACCCGGGTGGAATTCCTAGGCACATTTTGCGTT	
HMGR3-2300-Homologous arm-F	GCCATGGAGGCCAGTGAATTCATGGATCCTTTCCCCTG	
HMGR3-2300-Homologous arm-R	CCCACCCGGGTGGAATTCTTACATCTTTCCTTGTGAGCC	
AD-Kan-JD-F	TCGCAGATCTGTCGATCGACC	Identification of transgenic plants
AD-Kan-JD-R	CTCTAGCTAGAGGATCGATCCGAAC	

Table continues on next page

Table S5 (continued). Primer sequences

Primer	Sequence (5’→3’)	Function
DfHMGR1-SP1	CCGCTACGACAACCTCGAACAAG	Chromosome walking
DfHMGR1-SP2	CTACCATGTCTCCAAAGCTCAGGACAT	
DfHMGR1-SP3	GAGTTCTTGTTGCGCCTTGAATGC	
DfHMGR1-SP4	CCAAATCAAGGAGCATGTACTTCTTAAGGG	
DfHMGR1-SP5	ATGCTGTGACCAGTTACAGTCTTGGAT	
DfHMGR1-SP6	ATGCATGTTAGTTCGAGAGGAAGAGCT	
DfHMGR2-SP1	GCATCTGAGGCAAGGAGTTECACAT	
DfHMGR2-SP2	GTCTGCACCCATTGTGCAAAGCTC	
DfHMGR2-SP3	GAAGACTGGGTCTCTCAAGTTCGAGG	
DfHMGR2-SP4	GAAGACTGGGTCTCTCAAGTTCGAGG	
DfHMGR2-SP5	CGTGCGCGCATGTCTATGTATG	
DfHMGR2-SP6	CTCACATTGCCTACAGACGGT	
DfHMGR2-SP7	GCGTAAGATTGTGTGTGTGCCTACG	
DfHMGR2-SP8	CCACATACACGCAGATACGTAGTCAATG	
DfHMGR2-SP9	ACGAGATACATCCGATCTACAGAGAGC	
DfHMGR3-SP1	CATCGTCTGAGCAGGTAGTACGATGC	
DfHMGR3-SP2	AGCTCTTGACGCGTTTCATGCTG	
DfHMGR3-SP3	AGGAGAATGTAAGATGTGAAGATTGAAGGGAG	
FP1	GTAATACGACTCACTATAGGGCACGCGTGGT NTCGASTWTSGWGTT	
FP2	GTAATACGACTCACTATAGGGCACGCGTGGT NGTCGASWGANAWGAA	
FP3	GTAATACGACTCACTATAGGGCACGCGTGGT WGTGNAGWANCANAGA	
FP4	GTAATACGACTCACTATAGGGCACGCGTGGT AGWGNAGWANCAWAGG	
FP5	GTAATACGACTCACTATAGGGCACGCGTGGT NGTAWAASGTNTSCAA	
FP6	GTAATACGACTCACTATAGGGCACGCGTGGT NGACGASWGANAWGAC	
FP7	GTAATACGACTCACTATAGGGCACGCGTGGT NGACGASWGANAWGAA	
FP8	GTAATACGACTCACTATAGGGCACGCGTGGT GTNCGASWCANAWGTT	
FP9	GTAATACGACTCACTATAGGGCACGCGTGGT NCAGCTWSCTNTSCTT	
FP10	GTAATACGACTCACTATAGGGCACGCGTGGT TGWGNAGSANCASAGA	
FP11	GTAATACGACTCACTATAGGGCACGCGTGGT STTGNTASTNCTNTGC	
FP12	GTAATACGACTCACTATAGGGCACGCGTGGT TGAGNAGTANCAGAGA	
FP13	GTAATACGACTCACTATAGGGCACGCGTGGT AGTGNAGAANCAAAGG	
FP14	GTAATACGACTCACTATAGGGCACGCGTGGT CATCGNCNGANACGAA	
FP15	GTAATACGACTCACTATAGGGCACGCGTGGT TCGTNCGNACNTAGGA	
FP16	GTAATACGACTCACTATAGGGCACGCGTGGT CAWCGTCNGATASGGA	
FSP1	GTAATACGACTCACTATAGGGC	
FSP2	ACTATAGGGCACGCGTGGT	
Pro_{DfHMGR1}-full-F	GTCGACGGTATCGATAACTATAGGGCAACGCGTGGT	
Pro_{DfHMGR2}-full-F	GTCGACGGTATCGATACGTACACTCAAGCACGATTACG	

Table continues on next page

Table S5 (continued). Primer sequences

Primer	Sequence (5'→3')	Function
Pro$_{DfHMGR1}$-T1-F	GTCGACGGTATCGATAGTATATGTGTACATATGTACATAAATACAT	Promoter truncation
Pro$_{DfHMGR1}$-T2-F	GTCGACGGTATCGATAGTTTGTGCATCCTCACA	
Pro$_{DfHMGR1}$-T3-F	GTCGACGGTATCGATAACATGGAGTGATTGTCAAC	
Pro$_{DfHMGR1}$-T4-F	GTCGACGGTATCGATATCACAGCATTTTGACATATCC	
Pro$_{DfHMGR2}$-T1-F	GTCGACGGTATCGATACTACCCTAGGTGTGTGAG	
Pro$_{DfHMGR2}$-T2-F	GTCGACGGTATCGATATCACTTGTGGTCCTCAC	
Pro$_{DfHMGR2}$-T3-F	GTCGACGGTATCGATAACTTTCAGGGTGCAAAC	
Pro$_{DfHMGR2}$-T4-F	GTCGACGGTATCGATAGCCGGAGACCTTTATG	
Pro$_{DfHMGR3}$-T1-F	GTCGACGGTATCGATAAGAGAGAGCGTGTGTGA	
Pro$_{DfHMGR3}$-T2-F	GTCGACGGTATCGATAAAGTTGGCGCCCAAA	
Pro$_{DfHMGR3}$-T3-F	GTCGACGGTATCGATAATTCAATGAATGTGTGTGTTT	
Pro$_{DfHMGR3}$-T4-F	GTCGACGGTATCGATAACGCACACACCCATAC	
Pro$_{DfHMGR1}$-R	CAGGAATTCGATATCATGGTGTGACAAGCAAAAC	
Pro$_{DfHMGR2}$-R	CAGGAATTCGATATCATGTGCAAAGCTCAGCAATTCAAAATAAATCTGCCGGACAC	
Pro$_{DfHMGR3}$-R	CAGGAATTCGATATCAGGATAGAAGGAGAATGTAAGATG	

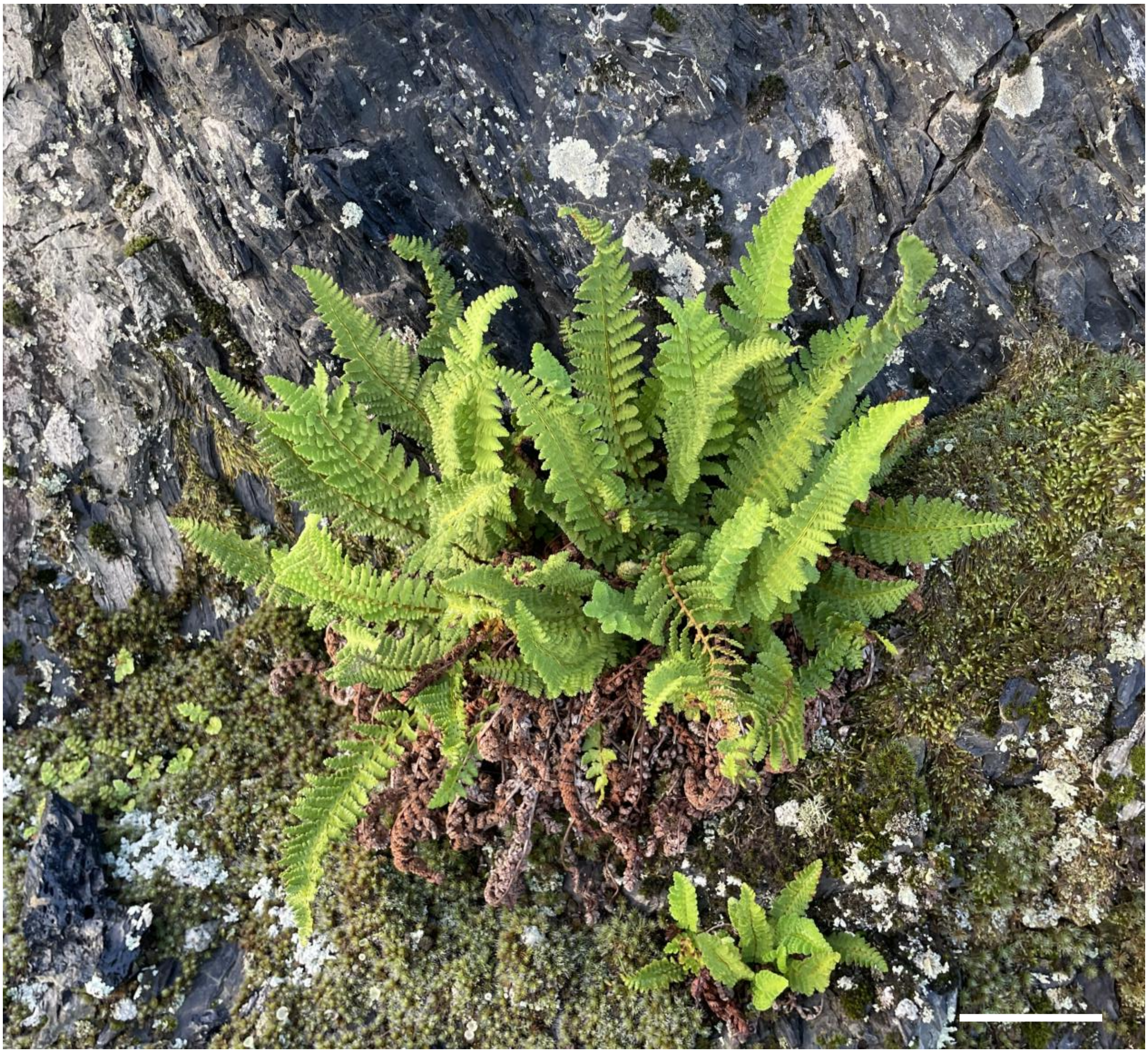

Figure S1. ***Dryopteris fragrans*** **collected from Wudalianchi, Heilongjiang Province, China. Bar = 5 cm.**

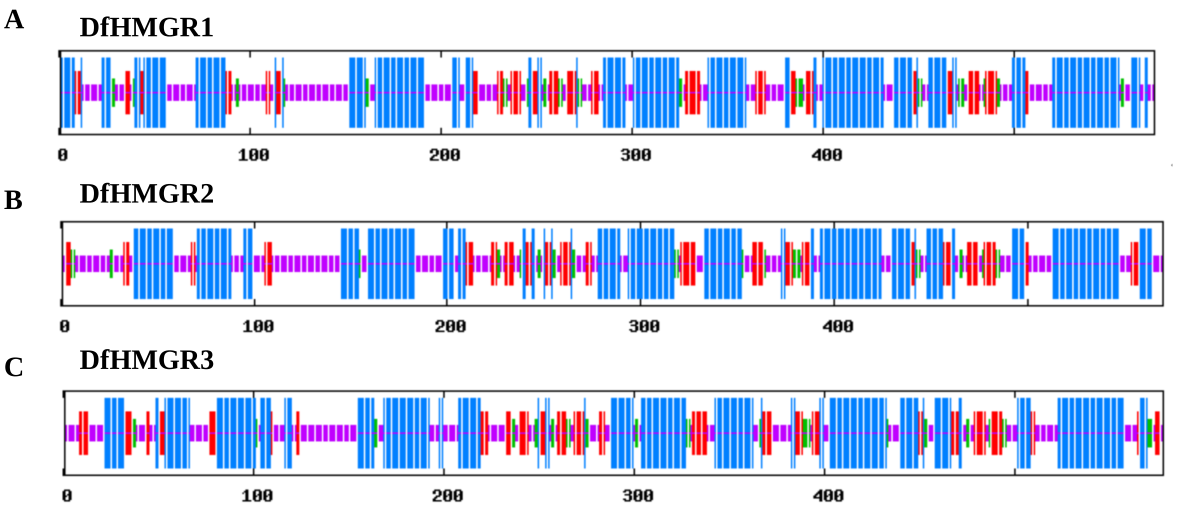

Figure S2. Secondary structure prediction of DfHMGRs. (A) DfHMGR1 secondary structure prediction results;(B) DfHMGR2 secondary structure prediction results;(C) DfHMGR3 secondary structure prediction results.

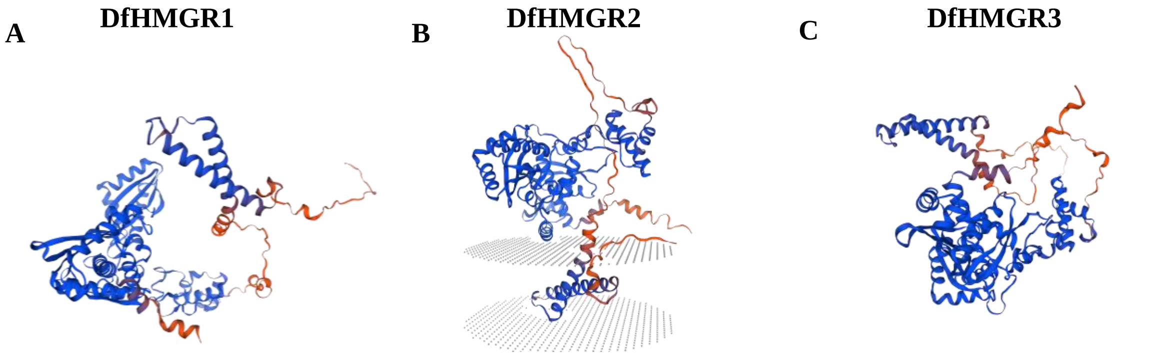

Figure S3. Tertiary structure prediction for DfHMGRs. (A) DfHMGR1 tertiary structure prediction results;(B) DfHMGR2 tertiary structure prediction results;(C) DfHMGR3 tertiary structure prediction results.

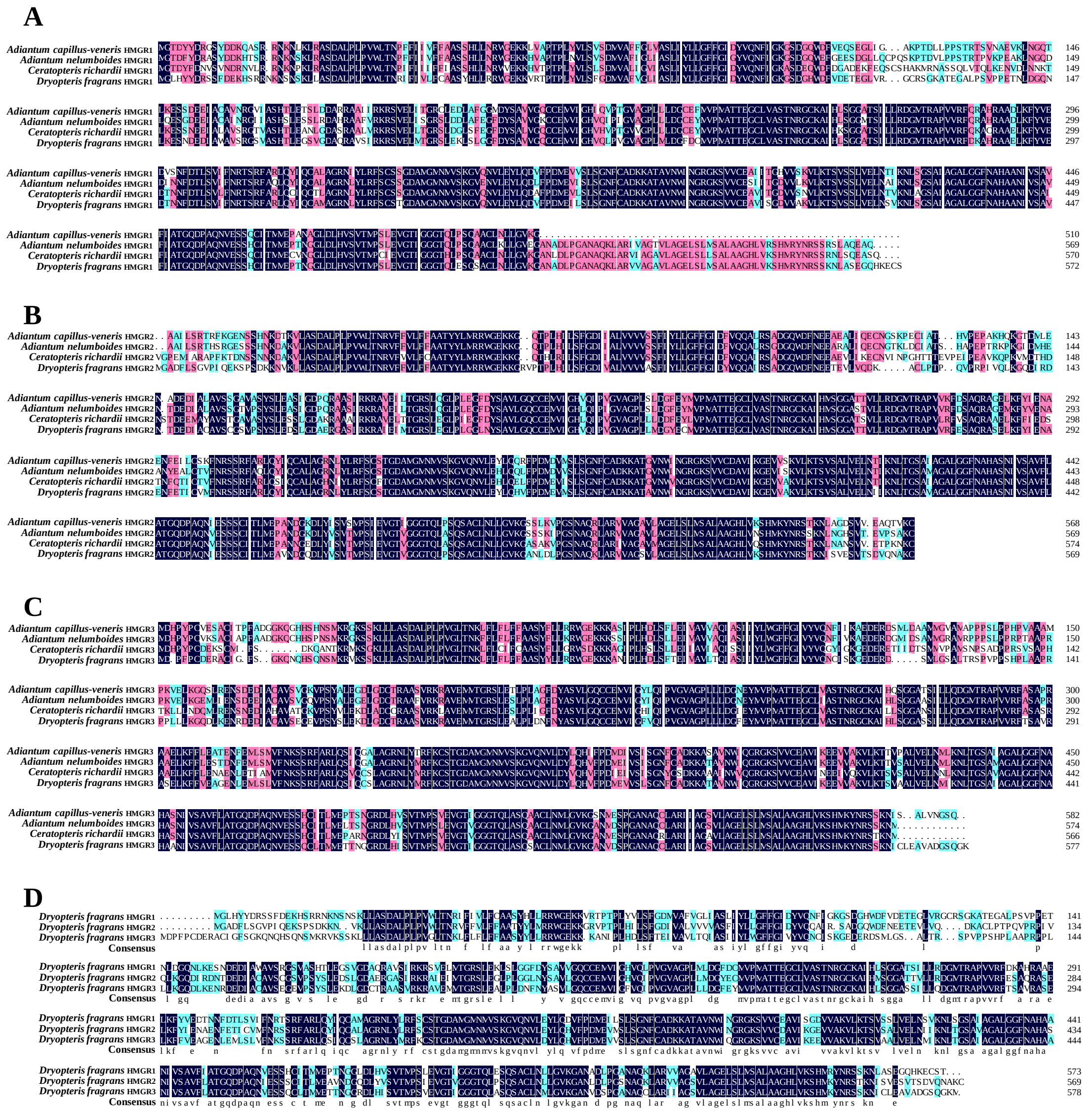

Figure S4. Results of multi-sequence alignment of DfHMGRs. (A-C) Multiple sequence alignment results of *Adiantum capillus-veneris*, *Adiantum nelumboides*, and *Ceratopteris richardii* with *Dryopteris fragrans* HMGRs;(D) Multiple sequence alignment results of *Dryopteris fragrans* HMGRs.

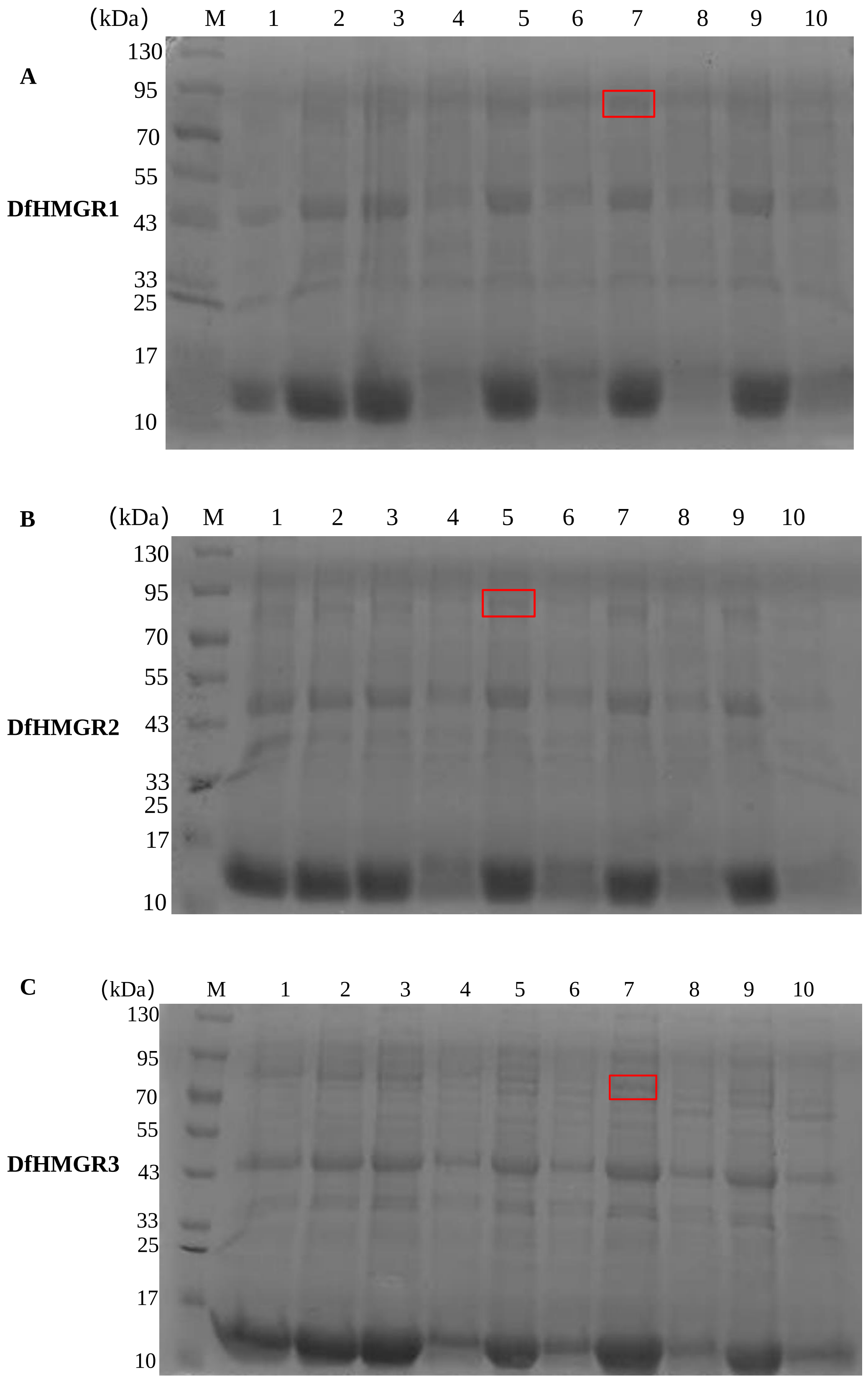

Figure S5. Inducible expression of DfHMGRs. (A) Results of induced expression of DfHMGR1;(B) Results of induced expression of DfHMGR2;(C) Results of induced expression of DfHMGR3. M: Protein marker; Lanes 1-10: Induced samples.

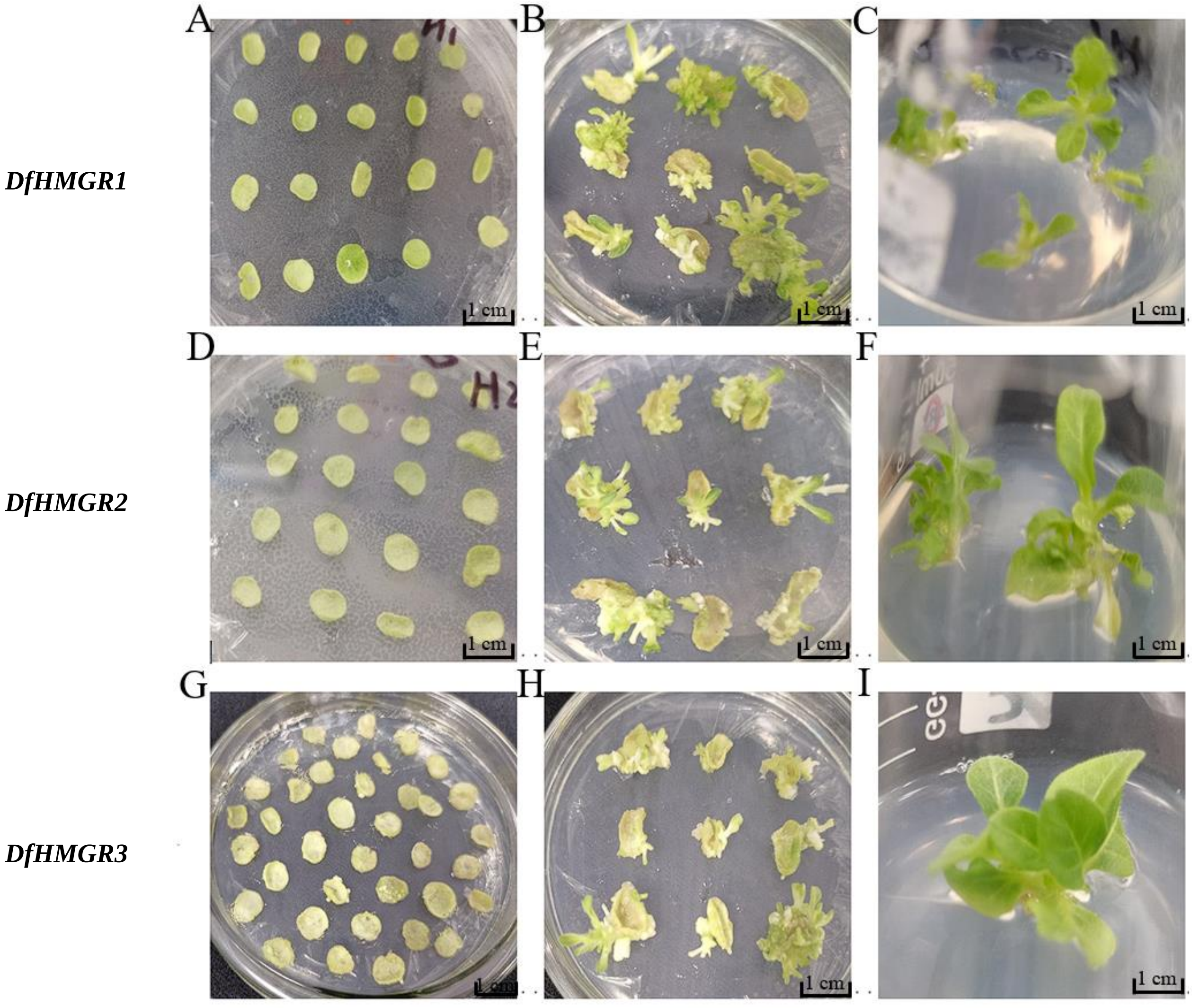

Figure S6. Generation of transgenic *N. tabacum* plants overexpressing *DfHMGR* genes. (A-C) Transformation process for *DfHMGR1* (A: Agrobacterium-infected leaf discs; B: Kanamycin-resistant shoot induction; C: Rooted transgenic plants); (D-F) Transformation process for *DfHMGR2* (D: Infected leaf discs; E: Resistant shoot induction; F: Rooted plants); (G-I) Transformation process for *DfHMGR3* (G: Infected leaf discs; H: Resistant shoot induction; I: Rooted plants). Scale bars=1 cm.

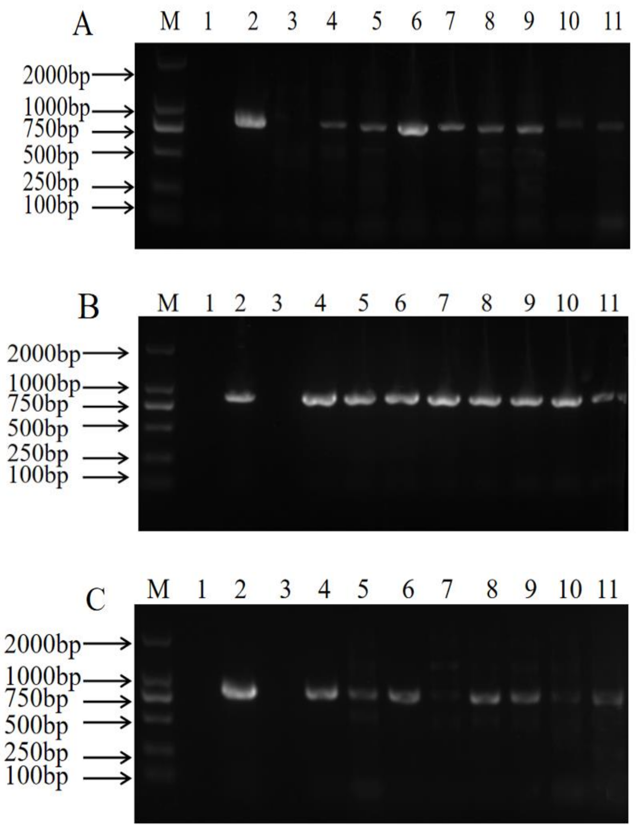

Figure S7. PCR identification of transgenic *N. tabacum* plants overexpressing *DfHMGRs*.
(A-C) PCR verification of *DfHMGR1*, *DfHMGR2* and *DfHMGR3* transgenic lines at DNA level (M: DL2000 Marker; lane 1: negative control; lane 2: positive control; lane 3: wild-type; lanes 4-11: transgenic resistant plants).
